# Artificial intelligence provides greater accuracy in the classification of modern and ancient bone surface modifications

**DOI:** 10.1038/s41598-020-75994-7

**Published:** 2020-11-02

**Authors:** Manuel Domínguez-Rodrigo, Gabriel Cifuentes-Alcobendas, Blanca Jiménez-García, Natalia Abellán, Marcos Pizarro-Monzo, Elia Organista, Enrique Baquedano

**Affiliations:** 1 Institute of Evolution in Africa (IDEA), Alcalá University, Covarrubias 36, 28010 Madrid, Spain; 2 Area of Prehistory (Department History and Philosophy), Alcalá de Henares University, Alcalá de Henares, Spain; 3 Prehistory Department, Complutense University, 28040 Madrid, Spain; 4 Osteoarchaeological Research Laboratory, Department of Archaeology and Classical Studies, Stockholm University, 106 91 WallenberglaboratorietStockholm, Sweden; 5 Museo Arqueologico Regional de la Comunidad de Madrid, Alcalá de Henares, Spain

**Keywords:** Anthropology, Archaeology, Palaeontology, Computational science

## Abstract

Bone surface modifications are foundational to the correct identification of hominin butchery traces in the archaeological record. Until present, no analytical technique existed that could provide objectivity, high accuracy, and an estimate of probability in the identification of multiple structurally-similar and dissimilar marks. Here, we present a major methodological breakthrough that incorporates these three elements using Artificial Intelligence (AI) through computer vision techniques, based on convolutional neural networks. This method, when applied to controlled experimental marks on bones, yielded the highest rate documented to date of accurate classification (92%) of cut, tooth and trampling marks. After testing this method experimentally, it was applied to published images of some important traces purportedly indicating a very ancient hominin presence in Africa, America and Europe. The preliminary results are supportive of interpretations of ancient butchery in some places, but not in others, and suggest that new analyses of these controversial marks should be done following the protocol described here to confirm or disprove these archaeological interpretations.

## Introduction

The correct identification of bone surface modifications (BSM) remains one of the most challenging topics in taphonomic research. Crucial interpretations on when humans started eating meat^1–3^, or using tools^4,5^, or colonizing new continents^6,7^ depend on correct identifications of BSM on fossil bones. In the past ten years, analytical tools for analyzing BSM have become very sophisticated, involving the use of 2D and 3D geometric morphometric analyses^8–14^, uni- and bivariate metric analyses through 3D reconstruction of BSM^15,16^, multivariate qualitative analysis using frequentist and bayesian traditional techniques^17–20^, machine learning analyses^21,22^, machine learning techniques combined with geometric morphometrics^23^ and, most recently, artificial-intelligence computer vision through deep learning (DL)^24^. All these techniques have increased our ability to combine multivariate information and classify marks with more certainty than using univariate approaches^21,25^. However, despite the higher efficiency of these methods in dealing with BSM information, some of them still rest on problematic foundations. Metric approaches have shown their inability to cope with large variations in single-agency samples: subsamples of the same original sample yield significant differences despite BSM having been created by the same agent^26^. Some of these methods have also not been tested to show high resolution at classification when using structurally similar types of BSM (e.g., cut marks *versus* trampling marks). Some geometric morphometric analyses have also overemphasized their success, using very small sample sizes and by showing accuracy rates that are similar to less sophisticated methods -e.g.,^27^. In addition, non-metric approaches rely on the categorization of variables by the analyst and this has been shown to be highly subjective^28^. Therefore, no completely objective method exists that efficiently uses information to provide highly accurate BSM classification rates.

Recently, this situation has been improved by the use of DL methods involving computer vision^24^. Automated DL methods capture features from BSM images and classify them according to trained models. Specific algorithms exist (e.g., Grad-CAM)^29^ which capture which features are used by DL models to identify each image type. This method removes subjectivity from variable information and uses a mega-dimensional framework to detect image features and associate them with specific categories. The success in accurate classification of BSM using this method (> 90%) with a limited number of marks has exceeded by 50% the correct assessment by experts^24^. This low accuracy by experts can also be explained in part by observing a mark at a time, instead of being able to apply a configurational approach, which would enable them to look simultaneously at associated marks and modifications on the same specimen. However, the same argument could be applied to the machine. Computer vision accuracy increases when the targeted object can be understood within its context. This reported high accuracy of DL models has led to test the method´s ability to identify categories that are structurally almost identical, by using samples of cut marks made with the same implements on fleshed and defleshed bones^30^. The DL algorithm classified successfully up to 95% of these structurally challenging BSM. In that study, the algorithm highlighted microscopic aspects of BSM that had not been detected before as relevant for discriminatory purposes. The machine, thus, returned information to human analysts, which was useful for their own training. These encouraging results support an artificial intelligence approach to the study of BSM.

These DL methods also enable the assessment of the probability of classification, as stressed by previous multivariate analytical studies^19,22^. Here, we will apply these DL techniques for the purpose of discriminating three types of BSM (cut marks, tooth marks and trampling marks). It has been argued that tooth marks are easily discerned from cut marks, given their widely divergent microscopic features^26^. It has also been emphasized that structurally similar BSM are more difficult to identify. Here, we will address these issues by comparing success in classification rates between structurally diverse BSM sets and structurally similar BSM samples. We will use a sample of tooth marks made by lions and wolves, cut marks made with simple stone flakes and, trampling marks from sand abrasion. Comparison between tooth marks and the other BSM will address accuracy in identifying structurally dissimilar marks. Comparisons between cut and trampling marks will focus on discerning structurally-similar marks. For the DL analysis, six different models combining simple and complex architectures will be implemented. Some of these models (Alexnet, ResNet50, VGG16, Inception V3) are winners of the Imagenet Large Scale Visual Recognition Challenge (LSVRC), the largest competition of image classification (1000 image categories), with accuracy rates > 90%. These models are used here as pre-trained architectures. This is complemented with two simpler models (Jason1 and 2), with the goal of showing that high accuracy can be reached not only with complex architectures, but also with simple ones. The use of bidimensional BSM images is challenging since most of these images are macroscopically very similar and only microscopic features identify them as belonging to specific BSM categories. Human taphonomists have been documented to exhibit an accuracy of 63% in differentiating these types of marks^24^. When using a multivariate set of microscopic variables, Bayesian and Fisherian discrimination methods have succeeded in classifying these and other similar marks correctly ranging from 68 to 80%^18,19^. However, these methods imply a high degree of subjective assessment of variables and are, therefore, subjected to the analyst experience^28^. Until present, no objective method (providing probability of classification) has achieved high accuracy in discriminating cut marks, tooth marks and trampling marks together. The DL method that we present here is the first one and it has yielded higher accuracy rates than any previous taphonomic approach in differentiating structurally similar and dissimilar BSM jointly. This provides a compelling referential base with which controversial cut marks in the archaeological record can be more solidly identified and interpreted. The application of this referential image database to some selected important BSM in the archaeological record provides some preliminary interpretations that challenge current interpretations on the earliest presence of humans in some major geographical areas and the earliest traces of butchery in the archaeological record.

## Results

**Figure 1:**
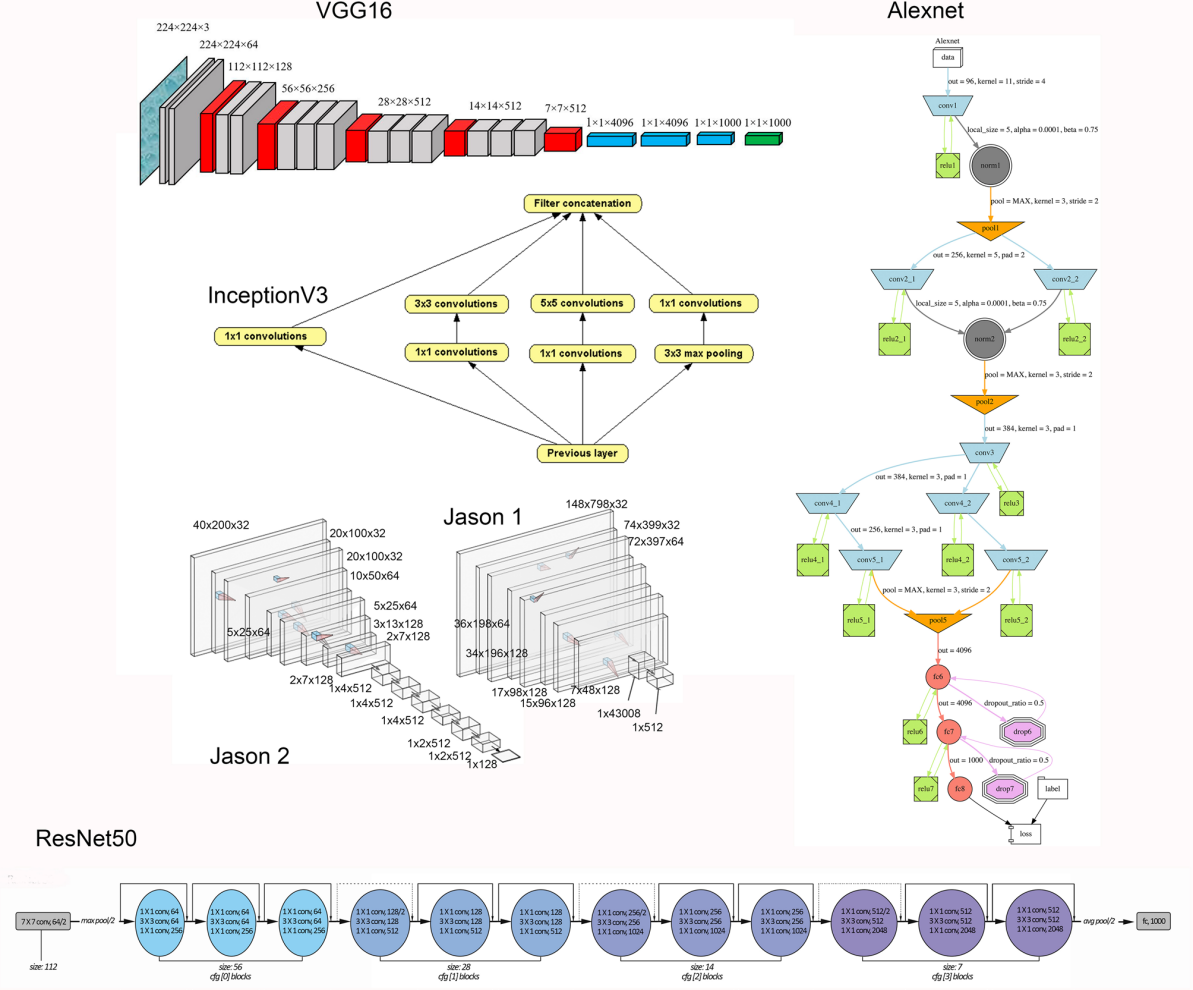
Architectures of the six models used to train the network (See parameter indication in Supplementary Information; Tables S1-S6). Image of VGG16 is by Nshafiei and is licensed under CC BY-SA 4.0. Image of Alexnet is by Miquel Perelló Nieto and is licensed under CC BY 4.0.

Each of the seven DL models tested provided a high accuracy in the classification of the three types of BSM of the testing set (Table 1). The lowest accuracy was provided by the pre-trained ResNet50 and InceptionV3 models (74%) and the highest one by the pre-trained VGG16 model (92%). Although the accuracy displayed by Alexnet was moderately good (78%), its loss was too large. The complex Jason2 model (accuracy = 0.86, loss = 0.57) did not yield a better accuracy than its simple version (Jason1; accuracy = 0.88, loss = 0.36). The two most stable models were VGG16 (Fig. 1) and Jason1 (with high accuracy and low loss). The low loss values of the highest ranking models also indicate a high probability of classification for individual marks. The pre-trained models performed unevenly according to the model selected (Table 1); however, the pre-trained architecture of VGG16 was substantially better than any of the other tested models (Table 2). This may result from the fact that the 1000 image categories of the 1.000.000-image training set contained much more complex features than the BSM images in the present study (mostly restricted to boundaries of grooves and internal features). To test this idea, we coded the VGG16 architecture and trained the model from the scratch using only our BSM image dataset. The result was substantially lower (accuracy = 76%). Therefore, we argue that the VGG16 architecture trained on the other extended image data set was specifically very adequate for the BSM problem at hand. In sum, our deep learning approach to identifying the three types of BSM successfully classified > 90% of the testing marks and did so with high probability for each mark.

**Table 1:** Accuracy and loss values for each of the six models tested.

Model	Accuracy	Loss
Alexnet	0.78	1.69
Jason1	0.88	0.36
Jason2	0.86	0.57
VGG16	**0.92**	**0.36**
ResNet50	0.74	0.96
InceptionV3	0.74	1.13
Densenet 201	0.76	0.76

**Table 2:** Classification report of the three types of bone surface modifications using the VGG16 model. Key: TM, tooth marks; CM; cut marks; TMP; trampling marks.

	Precision	Recall	f1-score	Support
TM	0.77	0.83	0.80	36
CM	0.94	1.00	0.97	152
TMP	0.70	0.30	0.42	23
Micro avg	0.90	0.90	0.90	211
Macro avg	0.80	0.71	0.73	211
Weighted avg	0.88	0.90	0.88	211

When documenting the classification of each of the three types of BSM using the VGG16 architecture, the recall is highest for cut marks (1.00), followed by tooth marks (0.83). On average, the classification accuracy is well balanced (F1-score = 0.73), with trampling marks showing a higher degree of misclassification (recall = 0.30) (Table 2). When misclassified, a greater number of trampling marks are classified as tooth marks instead of cut marks. This is advantageous for the preliminary analysis of archaeological BSM (see below), because it shows that: (a) the model excels at identifying cut marks; (b) the probability of misidentifying a cut mark for a trampling mark remains low, (c) the misidentification of a trampling marks for a tooth mark or viceversa has no archaeological relevance for the purpose of identifying anthropogenic agency and, (d) the identification of trampling in archaeological assemblages is reinforced by the fact that the model may sometimes identify a trampling mark as a cut mark but not the other way around.

Ensemble learning through model stacking yielded slightly lower results than the VGG16 model (Table 3). A base layer comprising all the transfer learning models used yielded an accuracy of 90% (F1 score = 0.71 with upper layer composed of a Random Forest and, F1 score = 0.73 with upper layer composed of a Gradient Boosted Machine). The accuracy was slightly lower when using models trained without augmentation (Table 3). The precision and recall information of these ensemble models are similar to that obtained for the VGG16 model.

**Table 3:** Classification report of the three types of bone surface modifications using the stacked ensemble learning analysis, divided by training type (with or without image augmentation), and basal and upper layer constitution.

	Basal layer	Upper layer	Accuracy	F1 score
With image augmentation				
	Jason2 VGG16 Resnet 50 Inception V3 Densenet 201	Random forest	0.90	0.71
		Gradient boosting machine	0.90	0.73
Without image augmentation				
	Jason2 VGG16 Resnet 50 Densenet 201	Random forest	0.88	0.73
		Gradient boosting machine	0.90	0.73

The present analysis suggests that for classifying bidimensional images of BSM, sequential models are more adequate than alternative computationally more complex residual and parallel architectures. The analysis also suggests that there are no panaceas for AI models. Ensemble learning is usually more compact and balanced than individual models, but sometimes single models can yield better results as is the case in the present study.

## Discussion and conclusions

Byeon et al.^24^ used a simple CNN model to correctly classify 91% of cut marks and trampling marks from a very small image bank consisting of only 79 experimental marks (42 trampling marks and 37 cut marks). These marks were highly selected prior to analysis and do not represent the complete range of mark features in each of the mark categories. The present study pretended to significantly increase the sample of marks used (n = 657 marks) and include a large range of mark morphologies and their associated features, which was missing in the previous analysis. We also wanted to test accuracy of the CNN models by adding an additional category of mark (tooth marks). Together, cut marks, trampling marks and tooth marks are the most widely studied BSM and the most important to derive interpretations of crucial paleoanthropological value from the archaeological record. The null hypothesis in the present study was that the CNN models would decrease their accuracy with respect to the baseline model provided by Byeon et al. given the augmented difficulty in identifying the extensively overlapping range of features and morphology of marks.

**Figure 2:**
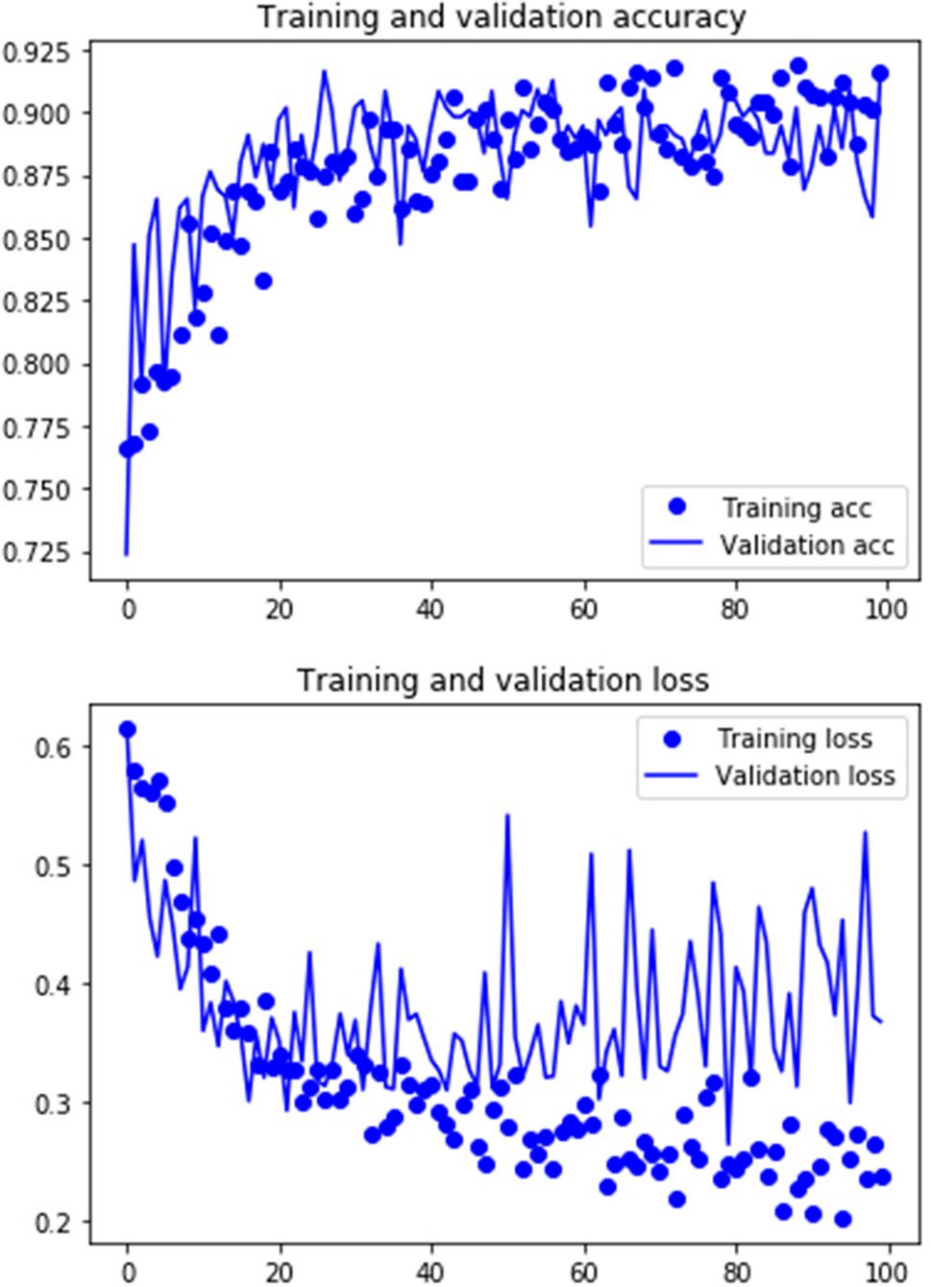
Percentage of accuracy and loss of the VGG16 pre-trained model along the 100-epoch sequence.

The results contradicted the null hypothesis by correctly classifying up to 92% of marks of the testing set. This was achieved with lower computation (100 epochs) than used by Byeon et al. (1000 epochs). This high accuracy in classifying these three types of marks has not been achieved before. Moderate to good classifications of BSM had been achieved with multivariate approaches to microscopic bidimensional and tridimensional features of marks^13,14,18–20^. Although confocal microscopy applied to cut marks and tooth marks reached a high accuracy^16,31^, no three-dimensional approach has provided any resolution to date in differentiating cut marks from trampling marks or small tooth marks from a certain type of trampling marks. The diversity of methods that use multivariate approaches has increasingly led to higher degrees of accuracy. The application of deep learning CNN is one of them, which leads not only to equally similar or even higher accuracy in BSM classification, but provides two advantages. One is that it is completely objective. The analyst has no input in tallying mark categories or during the classification process. This eliminates the subjective input of such an assessment^28^. The CNN objectively evaluates the complete image and extract identifying features. Secondly, the CNN models provide indications of model reliance. The optimizers used provide class probability and the loss estimates provide indication of model stability. The model with the highest accuracy provided also the lowest loss values and a low oscillation of the learning curve (Fig. 2). This is a sign of reliance and stability. The high accuracy and low loss reported here is not surprising. CNN models are able to discriminate images on the basis of extent of microscopic features such as flaking, microstriations and shoulder effects. A comparative analysis of cut marks made with the same tools on fleshed and defleshed bones showed similar rates of correct classification when tested through CNN models that were even simpler than those used here^30^. The resolution of these deep learning methods is such that they are capable of even differentiating cut mark modifications in one-minute sequences of exposure to trampling^32^.

We are aware that the results obtained here are based on the good preservation of the microscopic features of BSM. For this reason, the present dataset should only be applied to archaeofaunal assemblages where preservation is good. It could be argued that some diagnostic features of cut marks, such as the presence of internal microstriations, could disappear by biostratinomic processes (e.g., more than five minutes of intensive trampling) or diagenetic factors (e.g., chemical modification of BSM). These modifications can also affect trampling marks, making their distinction with cut marks even more challenging. In this case, the present dataset should not be applied. However, in those cases where either cut or trampling marks exhibit good preservation by documenting the presence of microstriations, the present dataset is adequate. We are also aware that the resolution of this framework can be improved by the enlargement of the experimental data sets, especially that of trampling marks. For a potential improvement in the reliability of identification of BSM on highly altered and chemically-modified bones, further experimental work applying this method is necessary. The controversial archaeological/paleontological BSM selected here for preliminary testing (Supplementary Information) exhibit good preservation, enabling the application of this method and its database. The resulting interpretations must be taken as strictly preliminary, since the images used were not obtained following the same methodological protocol as the reference images.

**Figure 3:**
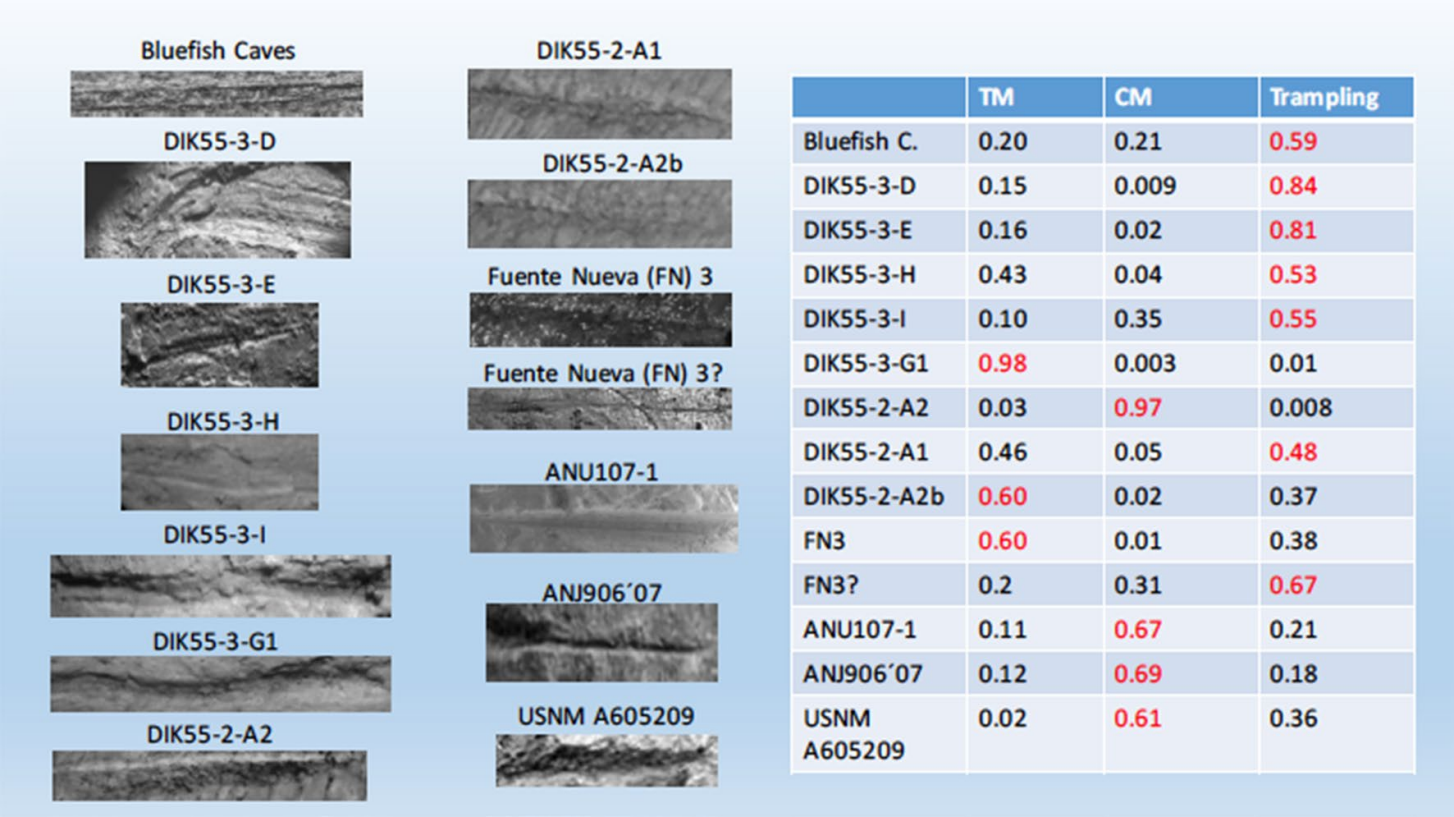
Selection of purported cut marks from controversial sites and classification by the pre-trained VGG16 model with probabilities (see description and sources of the images in Supplementary Information). Red indicates the classification result in each mark.

Given the combination of intensive training of the winning model over 1.000.000 images (and, therefore, the deep understanding of features) and the stability of the learning curve (high accuracy and low loss) over the re-training using hundreds of images of BSM, the model presented here is a solid referential framework to assess controversial BSM from archaeological contexts that exhibit good preservation. Its use on controversial marks from Arroyo Vizcaíno (Uruguay), the Cerutti Mastodon site or Blue Fish Cave (USA)^7,33,34^ should provide reliable assessment on the purported human nature of the modifications reported in those assemblages. Likewise, interpretations on controversial marks like those reported for the Plio-Pleistocene site of Quranwala^6,7^ in the Siwaliks (Pakistan) or the even older modifications reported at Dikika (Ethiopia) at 3.3 Ma^1,3^ could be further supported or rejected. Although images from BSM from these sites should be properly taken following the same protocols as in the experimental sample used here, the published images of some of these modifications provide preliminary interpretations when exposed to the CNN models presented here. For example, a selection of these controversial marks was tested against the classified VGG16 model (see Supplementary Information). One groove from a coxa (specimen #15.6.5) found at the 18 ka site of Bluefish cave (Alaska, USA) interpreted as a filleting cut mark^7,33,34^, is classified by the model as trampling mark (Fig. 3). Three BSM from the Anjohibe (2000–1400 B.C.)^35^, Itampolo (1800–1100 B.P.)^36^ and Christmas River (> 10.000 B.P.)^37^ sites (Madagascar) interpreted as anthropogenic are provisionally confirmed by the VGG16 model, although with moderate confidence (Fig. 3). This underscores the need to reanalyze these BSM with images obtained following the same protocol as the experimental dataset, because the model would provisionally suggest an older date for the human presence in Madagascar than posited by a recent thorough taphonomic review^36^. Likewise, two of the purported oldest cut marks in Europe^38^ fail to be confirmed by the model and are interpreted as a tooth mark and a trampling mark respectively (Fig. 3). This cautions on the heuristic of the interpretation of sabertooth felids being scavenging sources for early Pleistocene hominins, based on the interpretations of these marks.

A set of marks from the Dikika publication^1^ also provide diverse interpretations when tested through the model (Fig. 3) (Supplementary Information). Mark DIK55-3-D, interpreted by McPherron et al.^1^ as a cut mark, was classified here as trampling mark. DIK55-3-G1, interpreted by two of the three independent analysts in^1^ as “unidentifiable” and by another one as “cut mark”, was classified by the model as a tooth mark. Mark DIK55-3-E interpreted by the three analysts as a cut mark, was classified by the model as a trampling mark (with high confidence). Marks DIK55-3-H and DIK55-3-I, interpreted by McPherron et al. as percussion marks (or percussion mark + cutmark), were classified by the model as trampling marks. All these marks were previously also interpreted as probable trampling marks by^3^.

In contrast, mark DIK55-2-A2 (taken from McPherron et al.’s Fig. 2) was interpreted by the model as a cut mark (with high probability). However, this and the previous interpretations must be taken with caution because the images are in poor resolution and have been taken with different angles and magnification than specified for the experimental protocol. A proof of this is that marks DIK55-2-A1 and DIK55-2-A2 when taken from a different angle and magnification (McPherron et al.´s Supplementary Information, pag. 27) are classified by the model as a trampling mark and a tooth mark, respectively. Obviously, the poor resolution and pixelation of the images render these classifications unreliable. However, it is interesting that on the higher-quality image of DIK55-2-A2, the model feels confident in classifying the mark as a cut mark. Given the relevance of the Dikika fossil for human evolution, this emphasizes that proper high-resolution images of these marks are taken following the same protocol (including same magnification and zenithal angle) as described for the experimental sample reported here. It should also be emphasized that what the model is suggesting is that DIK55-2-A2 is not a trampling mark made with sand, which is the main knowledge the model has about trampling, since most of the trampling marks used were derived from sand abrasion and substantially fewer were from gravel. Domínguez-Rodrigo et al.^3^ interpreted the two DIK55-2 marks as “morphologically compelling in their similarity to verified cut marks created by stone tools used in experimental butcheries: the marks show deep, V-shaped cross-sections and contain microstriations. In a less contentious context, the marks would likely be accepted as genuine cut marks. However, the prominence of high-probability trampling damage (described earlier) on both DIK-55–2 and DIK-55–3 casts doubt on that diagnosis in this case—a geologically coarse-grain context older than the earliest known stone tools”. Given the presence of gravel in the sequence where the Dikika fossils were found and the replication of similar marks when trampling was made on gravel^3^, trampling made on fine and medium-sized sand is not a good proxy. For this reason, the very limited trampling image database using reported in the present study should be significantly increased with more images of marks made in systematic trampling experiments using gravel, instead of mostly sand. It will be interesting to test then (a) if the model still exhibits high accuracy in the presence of several (i.e., more than two) structurally-similar types of BSM and, (b) if the preliminary assessment of the archaeological marks reported here still holds or need to be modified. One way or another, the results will be of paramount importance for human evolutionary studies.

## Sample and method

### Sample

BSM were obtained from three different experiments. For tooth marks, a combined sample of lion-inflicted and wolf-inflicted tooth marks was used. The lion tooth mark sample was obtained from the experiment reported by Gidna^39^, carried out with a group of semi-captive lions from the Cabárceno (Cantabria) reserve in Spain and from the modern Olduvai Carnivore Site (OCS)(Tanzania) reported by Arriaza et al.^40^. Equid bones belonging to the four meat-bearing long limb elements were used for feeding lions at Cabárceno. The OCS sample is composed exclusively of wildebeest bones. The wolf tooth mark sample was obtained from a collection of long bones consumed exclusively by wolves in the reserve of El Hosquillo (Cuenca, Spain). These bones were composed of cervid and ovicaprid elements. Bones from the lion feeding experiment were cleaned by boiling them in a solution of water and neutral soap and then letting them dry up. Bones from the wolf experiment were cleaned following the same protocol. The original collection of tooth marks from both experiments was 208 tooth marks from lions and 210 tooth marks from wolves. However, given that a large number of tooth marks was documented on curved surfaces, some of them suffered from distortion and non-focused areas on the lateral sides. Because of this, many of these marks were discarded for the analysis and only well-focused images of individual marks were used (n = 106). All the tooth marks used in the present analysis are tooth scores. Tooth pits were also documented and photographed but they were not included in the study.

For the cut mark experiment, a set of cow long bones (*humerus*, *femur*, *radius* and *tibia*) were used along with 22 non-retouched flint flakes (see Supplementary material). Stone tools were used to mark fresh bone flesh-bearing shafts during butchery. Carcasses were butchered in a period less than a week from acquisition for all the cut-mark sample. Important differences in the micro-features of cut marks were previously discovered when using experimental cut marks imparted on fleshed versus defleshed bones^30^. Thus, fleshed bones are a more reliable proxy when aiming to reproduce cut-mark morphologies produced during butchery and bulk defleshing. Each stone tool was used only 20 times to keep control of edge sharpness and by doing so, ensuring that this did not play any significant role in possible cut-mark variability. Latter, bones were cleaned by submerging them in a solution of neutral detergent and boiling water during a complete hour. A deep inspection of bone surface and mark features followed to test whether or not the cleaning process might have altered mark features. Since shoulder flaking and internal micro-striations (which are the features most prone to be deleted by any diagenetic or biostratinomic processes, see^18^ were well preserved, we assumed that the cleaning process did not interfere with the original properties of the marks. The protocol was performed in accordance with the relevant guidelines and regulations. Bones and fleshed carcass parts were obtained from a commercial butcher, which complies with regulations according to the Spanish Ministry of Health. The experiments were conducted following the approval and protocols implemented in the Institute of Evolution in Africa.

Trampling marks were obtained from two separate experiments. Both have been reported independently. One experiment targeted the study of cut mark dynamic modification when exposed to trampling^32^. The experiment methodology used two variables: trampling time and the sediment type. Two types of sediment were selected: sand with a size of grain < 2 mm, and small gravel spanning in size from 2 mm to 1 cm. Trampling was carried out by laying each bone on the sediment and for a duration of one minute at a time. Given the non-compact nature of sediment, bones moved from their surficial position to a depth of 10–15 cm. Trampling was made by a shod 70-kg individual stepping on the bones. The other experiment documented the micromorphological features that served to discriminate trampling marks from cut marks and was reported by Domínguez-Rodrigo et al.^18^. Five sediment types were selected: fine-grained sand (0.06–0.2 mm), medium-grained sand (0.2–0.6 mm), coarse-grained sand (0.6–2.0 mm), a combination of the previous sand types over a clay substratum, and gravel (> 2.0 mm). In each sedimentary context, trampling was carried out in two experimental sets with different times, reproducing brief (10 s) and prolonged (2 min) exposure to trampling. Trampling was made by three experimenters (with different body weights ranging from 52 to 80 kgs) wearing shoes with esparto grass soles. Bones from deer (long bones and ribs) were used for the trampling experiment. Although more than 200 individual marks were photographed from both experiments together, a thorough screening of image quality detected some distortion between the interior of the grooves and the cortical surface due to the limited depth of field of the equipment used. This is why a large part of the original image database was not included in the present study.

Individual BSM were documented with a binocular microscope (Optika) at 30 X and images were taken in this magnification using the same light intensity and angle. Then, images were cropped to a point were only the mark and their shoulders were visible, to avoid any bias potentially produced by the cortical surface of the bone. The resulting image data bank (composed of 488 cut marks from the single-flake experiments, 106 tooth marks from experimental work with lions and wolves, plus 63 marks from trampling experiments) was used for analysis through the DCNN models described below.

All images were transformed into black and white during image processing in the Keras platform, by using bidimensional matrices for standardization and centering, and they were reshaped to the same dimensions (80 × 400 pixels).

It could be argued that we only experimentally reproduced a limited set of variables compared to the much more complex and palimpsestic array of processes and agencies that frequently impact archaeological BSM. Although tooth marks might eventually be differentiated among carnivore agents, it is commonly agreed that tooth marks are structurally similar (i.e., U-shaped sections, symmetric trajectories, internal flaking and/or polishing) and differentiable from BSM generated by abrasive processes (i.e., by angular walls, different groove sections, internal microstriations, shoulder effects). Therefore, by combining a strict flesh eater (lion) with a durophagous carnivore (wolf), we combine the moderate and intensive modifications created by both types of carnivores, which could also be represented in other similarly defleshing (e.g., cheetah) and durophagous (e.g., hyena) taxa. This provides a good diversity of tooth marks. Likewise, by selecting five different types of sediment, our experimental sample embodies different shapes and sizes of trampling marks, which mimic the most commonly represented trampling mark spectrum in archaeofaunal assemblages, since these sediments are similar to those represented in a large part of archaeological sites. Given that our primary interest is early Pleistocene archaeology, we focused on simple flakes for the elaboration of cut marks. Simple flakes are also the most commonly used tool for butchery across all the Pleistocene. Future work should emphasize adding other less common butchering tools like retouched flakes and even handaxes. Although the number of processes and agents involved in our experimental sample is limited (to enhance better control of the causal process), it certainly represents the most common taphonomic factors found in archaeofaunal assemblages: hominin impact (cut marks), carnivore modification (tooth marks) and sedimentary abrasion (trampling).

Given that all our experimental collections were made on fresh bones with limited biostratinomic exposure and no diagenetic modification, they are most aptly analogous to archaeological assemblages that exhibit good faunal preservation and no weathering (either subaerial or chemical). Future experimental work can expand this referential collection by adding images from BSM (including the three types of the present study) modified longitudinally by physical and chemical processes. These are irrelevant for the present manuscript because the archaeological marks selected are from specimens unaffected by these factors in an intense way.

### Method

The DL method used here involves models based on convolutional neural networks (CNN). For a detailed description of CNN see^41,42^. An in-depth description of the tuning of parameters used in CNN can be found in^43^. The CNN architectures used here were elaborated using the Keras platform with a Tensorflow backend. Computation was carried out on a GPU HP Workstation. Several model architectures were tested and compared (see below). The CNN models tested were made with the sequential and functional Keras API.

The model architectures tested were as described by^44^:

#### Alexnet

This model competed in the Imagenet Large Scale Visual Recognition Challenge (LSVRC), the largest competition of image classification, and was the winner in 2012 by achieving an error of 15%^45^. Alexnet consists of a sequential model of eight layers. The first five layers contain three convolutional layers alternating with two max-pooling layers topped by three successive fully connected layers. Max-pooling layers are used to downsample the dimensions of the tensors while keeping the depth constant. The last convolutional layer is followed by an overlapping max-pooling layer leading to two fully connected layers which feed into a softmax classifier. The model contains 96 3 × 3 kernel filters which are used with a 2 × 2 stride. The authors minimized overfitting by implementing image augmentation through image mirroring and randomly cropping. They also used Dropout techniques^46^. Dropout involves dropping neurons randomly during the forward and backward propagation process. This results in strengthening the weight parameters and reduces the chances of overfitting. The model contains 60 million parameters. The full model is displayed in Fig. 1 and Table S1.

#### Jason1

This model is inspired on the VGG architectures using a reduced version of the model suggested by^47^. In this version, we used eight layers with weights (4 CNN and 4 max-pooling layers). In the first CNN layer we used 32 filters, followed by a layer of 64 filters and a double layer of 128 filters, each containing 3 × 3 kernels. In between, we used 4 max-pooling layers with 2 × 2 kernels. The sequence was flattened and filtered through a dense layer of 512 neurons with an output activated via softmax. The full model is displayed in Fig. 1 and Table S2.

#### Jason2

This net represents and expansion of the Jason1 architecture and incorporates a display of regularization and overfitting-control techniques. It is a more complex model. The architecture represents a variant of the VGG16 block and repeated layer structure. As described in^30,32^, the model consists of a series of three blocks, each of them containing 3 × 3 kernel double layers of 32, 64 and 128 neurons respectively. In between each block, there are max-pooling (2 × 2 kernel) layers. Batch normalization has been applied to all the blocks. Additionally, Dropout has been implemented with increasing proportion (0.2, 0.3 and 0.4). At the end of the network, flattening was performed and a dense layer (128 filters) has been added. This was followed by a 0.5 Dropout layer and topped by a dense layer with “softmax” activation. Each CNN has been tuned with a “He uniform” initializer and padding. Stochastic Gradient Descend was the used optimizer with a learning rate of 0.001 and a momentum of 0.9. The model is displayed in Fig. 1 and Table S3.

#### VGG16

The famous VGG-16 and VGG-19 architectures were also winners of the Imagenet international competition in 2014^48,49^. The VGG-16 architecture had more than 138 million parameters. The model originally contained 16 layers with weights, organized in a series of 3 × 3 kernel CNN piled on top of each other with increasing depth, spanning from 64 to 512 filters in duplicated sequences. An extension was made with VGG-19 including 19 weighted layers. Matrix size was reduced using max-pooling layers in-between neural layers. The full model is displayed in Fig. 1 and Table S4.

#### ResNet50

This is a deep residual CNN, containing 50 layers. It was the winner of the 2015 LSVRC context with only 3.5% error in classifying the ImageNet testing set^50^. The architecture contains residual functions that allow the training of extremely deep models. These identity functions are used via a skip connection that allows to pass the input through blocks without having to pass through weight layers, thus coping with the problem of vanishing gradient that affects deep architectures. This makes it possible to train residual CNN of more than 100 layers. The model expands the VGG repeated layer blocks. Each block is three-layer deep. The initial CNN has 64 7 × 7 kernel filters and is followed by a max-pooling 2 × 2 kernel layer. Then, there is a block of three layers: one containing 64 1 × 1 kernel filters, one containing 64 3 × 3 kernel filters and one composed of 256 1 × 1 kernel filters. This block is repeated three times. Then a new series of CNN is followed composed of four blocks of two 128 filter units and one of 512 units, with the same filter size as in the previous block. This series is followed by another one composed of six blocks of two 256-filter layers and one 1024-filter layer. The last series contains three blocks of three layers each (two units with 512 filters and one with 2048 filters). This is topped with an average pooling layer and a fully connected layer. The full model is displayed in Fig. 1 and Table S5.

#### InceptionV3

This net has a similar degree of complexity to VGGNet, but with substantially fewer parameters^51^. It was initially labelled GoogleLeNet. Several versions have been implemented. the model reached the third position in the LSVRC contest in 2015 with a similar error rate as ResNet50. The net is structured around 42 layers. Inception computes these layers in parallel modules. The structure is based on a 5 × module, a 4 × module and a 2 × module separated by grid-size reduction modules. All of them contain CNN, average pooling layers (max-pooling layers in the grid-size reduction modules), concatenation units and Dropout. It uses factorization, a technique that reduces filter sizes and the number of parameters. This reduces the chances of overfitting and enables the network to proceed deeper. The model also uses one auxiliary classifier consisting of one average pooling layer of 5 × 5 (stride of 3), one 768 (5 × 5 kernel) layer, one 128 (5 × 5 kernel) convolutional layer and a fully connected 1024 (1 × 1 kernel) layer. Batch normalization is used in the classifier. The web structure of the net and the full model are displayed in Fig. 1 and Table S6.

#### Densenet 201

This is a fairly deep architecture consisting of 201 layers. Each sequential layers gets the feature maps of all the preceding layers as inputs and the resulting feature maps are passed subsequently to all the following layers. This renders the network thinner and more compact. This structure, combined with the depth of sequential CNN layers, enables the detection of a wider diversity of features in images compared to other alternative architectures. The model is structured around dense blocks CNN consisting of 1 × 1 and 3 × 3 sequential CNN layers, separated by transition blocks composed of 1 × 1 CNN and 2 × 2 pooling layers. The sequence of CNN in each dense block is repeated 6 times for the first block, 12 times for the second, 24, 32, 48 and 64 times for the third block and 16,32 and 48 times for the last block. The last transitional layer is a 7 × 7 global average pooling layer.

For all the models used, the activation function for each layer was a rectified linear unit (ReLU). Both this function and its combinations are non linear. The last fully connected layer of the network used a “softmax” activation. This function is specific for multinomial classification. It yields the probabilities of each input element of pertaining to a specific label.

The loss function selected was categorical cross-entropy, adequate for multi-class comparisons. Cross-entropy measures distances between probability distributions and predictions^41^. Tests combining different optimizers (including Adam) were made. A comprehensive comparison of multiple optimizers for CNN models supports the use of Adam over the rest^52^; however, here, the “SGD” optimizer was selected for all models because it provided better results. Learning rate was placed at 1e-3. Accuracy was the metric selected for the compilation process^47^.

The architectures were trained on about 70% of the original image set. The resulting models were subsequently tested against the 30% remaining sample, which was not used during the training. Training and testing were performed through mini-batch kernels (size = 32). Pairwise comparisons were made using a backpropagation process for 100 epochs.

Data augmentation was used to avoid overfitting and to artificially enlarge the sample. This method is highly recommended for small sample sizes, since it increases its heuristics of the neural architecture^41^. In this study, samples were augmented via random transformations of the original images involving shifts in width and height (20%), in shear and zoom range (20%), and also including horizontal flipping, as well as a rotation range of 40º.

All the model architectures were coded from the scratch, except for VGG16, ResNet50, DenseNet 201 and InceptionV3, which were used via transfer learning. Transfer learning consists of using a model to a specific problem that was trained for a different problem^43^. When the original image problem for which the model was trained was complex, the complexity of the neural network may even be more resolutive on the new problem than the same model run from the scratch, because it was trained for more complex features. Here, the most high-performing models trained on more than 1.000.000 images for the 1000-image category ILSVRC competition were used. These pre-trained models were used as standalone feature extractors and classifiers. The layers of the pre-trained models with their weights were integrated within the new model containing an output dense layer containing 128 neurons. This was implemented through the Keras API. The models selected are complex and were trained over such a large database that running them from the scratch would have required large computation power. By using them in the form of pre-trained models, training of the new model including the BSM images was much more efficient than performed only on the BSM image database and computation was much faster.

As a complement, ensemble learning via stacking ensemble was implemented. This technique allows to use the scores of individual models to generate a more comprehensive collective meta-analysis, resulting in more accurate and balanced models. Here, four transfer learning models (VGG16, Resnet50, Inception V3, Densenet 201) and the Jason 2 model were used on the image augmentation trained algorithms. Then, the same algorithms were trained without image augmentation and four transfer models were used as an additional comparison. In the latter case, Inception V3 was discarded because it yielded substantially lower accuracy and it might have pulled down the performance of the ensemble analysis. These models were used as the basal layer. Then they were ensembled via two different algorithms in the upper layer: a Random Forest (n trees = 100) and a Gradient Boosting Machine. The latter was tuned with a learning rate of 0.05, loss determined by “deviance” and 500 estimators.

## Supplementary information

Supplementary Information.
