# Author Correction: Artificial intelligence provides greater accuracy in the classification of modern and ancient bone surface modifications

**DOI:** 10.1038/s41598-021-82916-8

**Published:** 2021-02-08

**Authors:** Manuel Domínguez‑Rodrigo, Gabriel Cifuentes‑Alcobendas, Blanca Jiménez‑García, Natalia Abellán, Marcos Pizarro‑Monzo, Elia Organista, Enrique Baquedano

**Affiliations:** 1 Institute of Evolution in Africa (IDEA), Alcalá University, Covarrubias 36, 28010 Madrid, Spain; 2 Area of Prehistory (Department History and Philosophy), Alcalá de Henares University, Alcalá de Henares, Spain; 3 Prehistory Department, Complutense University, 28040 Madrid, Spain; 4 Osteoarchaeological Research Laboratory, Department of Archaeology and Classical Studies, Stockholm University, 106 91 Wallenberglaboratoriet Stockholm, Sweden; 5 Museo Arqueologico Regional de La Comunidad de Madrid, Alcalá de Henares, Spain

Correction to: *Scientific Reports* 10.1038/s41598-020-75994-7, published online 02 November 2020

The original version of this Article contained errors. The location of the Blue Fish Cave was incorrectly listed as the USA; the cave is located in Canada. Additionally, References 7 and 34 were incorrectly used to support the location of this site. This is now corrected and in the Discussion and Conclusions.

“Its use on controversial marks from Arroyo Vizcaíno (Uruguay), the Cerutti Mastodon site or Blue Fish Cave (USA)^7,33,34^ should provide reliable assessment on the purported human nature of the modifications reported in those assemblages.”

now reads:

“Its use on controversial marks from Arroyo Vizcaíno (Uruguay), the Cerutti Mastodon site (USA) or Blue Fish Cave (Canada) ^7,33,34^ should provide reliable assessment on the purported human nature of the modifications reported in those assemblages.”

and

“One groove from a coxa (specimen #15.6.5) found at the 18 ka site of Bluefish cave (Alaska, USA) interpreted as a filleting cut mark^7,33,34^, is classified by the model as trampling mark (Fig. 3).”

These references are now removed and the sentence reads:

“One groove from a coxa (specimen #15.6.5) found at the 18 ka site of Bluefish cave (Yukon, Canada) interpreted as a filleting cut mark^33^, is classified by the model as trampling mark (Fig. 3).”

Finally, the Supplementary Information file inaccurately reported that faunal assemblage is composed of 3,600 bone specimens. The assemblage is composed of 36,000 bone specimens.

These errors have been corrected in the HTML and PDF versions of the Article and in the Supplementary Information file.

