# Supplementary material for: Artificial intelligence provides greater accuracy in the classification of modern and ancient bone surface modifications
Source: Sci Rep. 2020 Nov 2;10:18862. doi: 10.1038/s41598-020-75994-7 (PMC7606445; doi:10.1038/s41598-020-75994-7)
Supplement: Supplementary file 1 — Supplementary Information. [file 41598_2020_75994_MOESM1_ESM.pdf]

SUPPLEMENTARY INFORMATION

Artificial intelligence provides greater accuracy in the classification of modern and ancient bone surface modifications.

Manuel Domínguez-Rodrigo[1,2], Gabriel Cifuentes-Alcobendas[1], Blanca Jiménez-García[1], Natalia Abellán[1], Marcos Pizarro-Monzo[1], Elia Organista[1,3], Enrique Baquedano[1]

[1]Institute of Evolution in Africa (IDEA), Alcalá University, Covarrubias 36, 28010 Madrid, Spain.
[2]Area of Prehistory (Department History and Philosophy), Alcalá de Henares University, Alcalá de Henares, Spain.
[3]Osteoarchaeological Research Laboratory, Department of Archaeology and Classical Studies, Stockholm University, Wallenberglaboratoriet, SE-106 91, Stockholm, Sweden.

Index

1. Archaeological cases and their contextual information
    a. Bluefish Caves (Alaska, USA)…….. page 2
    b. Dikika (Ethiopia)……………………..... page 3
    c. Madagascar…………………………. page 7
    d. Orce (Spain)…………………………. page 9

2. Model architectural parameters …………… page 16
3. Tools used in the experiment……………… page 33

1. Archaeological cases and their contextual information

1a. Bluefish Caves (Alaska, USA)

This set of three karstic caves contains some bone accumulations dating back to more than 14.000 years (which is the datum traditionally assumed to indicate the earliest presence of humans in the Americas), some of which could have been modified by humans. Bones exhibit green fractures implying a substantial amount of either dynamic or static loading. No percussion marks have been identified. Tooth marks are the predominant type of bone surface modification.

The accumulation of the faunal assemblages has been interpreted as the result of the behavior of lions, wolves and foxes (1). Today, only foxes out of these agents have been documented to systematically make accumulations of small game and mesofauna. This contrast with the fauna represented at the site, composed of large game (e.g., caribou, horse, mammoth), requires a bigger-sized accumulating agent. Although lions have been exceptionally interpreted as accumulators of bones in certain specific ecological conditions (nomads exposed to the competition of prides and other carnivores) (2), usually they do not transport carcasses systematically to the same loci. The same has been argued for wolves, who usually consume their prey on the kill spots (3). This casts some uncertainties about agency and the faunal assemblages found at Bluefish caves.This increases the chances that humans could have been an accumulation agent, given the presence of lithics on parts of the deposit. Carnivores seem to have been the main modifying agent given the high percentage of tooth-marked bones spanning from 14% to 58% of the assemblages. Out of more than 3600 bone specimens, 15 specimens were interpreted as bearing cut marks (1).

Recent dating of the faunal remains purportedly bearing anthropogenic marks (an equid metatarsal with cutmarks) yielded ages older than 17.000 years (1), thus questioning the standard earliest dates of human arrival in America of 14.000 years ago. Dating of a caribou innominate and an equid mandible also bearing purported cut marks has pushed even further back in time these dates to 22.000-24.000 B.P. If true, this would modify our current concept on the earliest human presence on the American continent. For this reason, the cut mark reported on the caribou

pelvic specimen was selected for analysis, given its good preservation compared to the equid mandibular specimen. The equid mandible surface exhibit intensive biochemical modification caused by root etching, which distorts the microscopic features as identified by our computer vision model and can easily yield biased accuracy in its identification. Results are discussed in the main text. The only mark analysed was interpreted as a trampling mark. However, this does not preclude that most of the other bone surface modifications (BSM) interpreted as cut marks could indeed be anthropogenic.

BSM selected from (1) for analysis (modified from Figure 2 in the original publication):

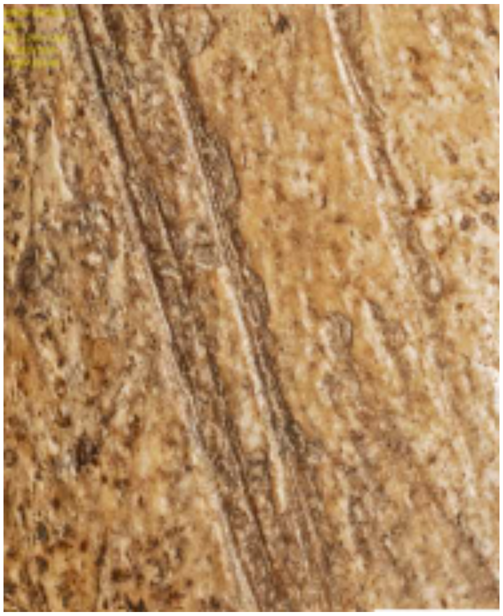

1b. Dikika (Ethiopia)

Two fossil specimens (DIK55-2 and DIK55-3) bearing up to 12 clearly identifiable bone surface modifications (BSM) were found on surface at the Pliocene locality of Dikika, north of Ethiopia (4). These BSM were independently assessed by three analysts, leading them to interpret most of them as cutmarks and percussion marks. A critical review of the modifications observed at the microscopic level led to the interpretation of ten out of these BSM as probably trampling marks and two as ambiguous (5). McPherron et al. interpreted the purported cutmarks as caused by the use of natural rock edges instead of flaked artefacts and blamed criticism to paradigm defense (6). Subsequent experimental butchery undertaken with natural rocks further questioned that the Dikika modifications could be interpreted as butchery marks (7) and that criticism had nothing to do with paradigm dogmatism but with lack of unambiguous empirical support (8). The subsequent discovery of Lomekwi-3 in Kenya, a “Pliocene” artefact assemblage composed of Oldowan flaked tools combined with ambiguously flaked stones, was used as a reinforcement argument for the interpretation of use of stone tools and butchery in the Pliocene (9). Shortly after the report, the in situ nature of the Lomekwi artefacts was put in doubt, since the original publication did not have any secure identification of artefacts in Pliocene sediments (10). Subsequent information about the Lomekwi-3 excavation (11) provided more fuel to skeptical

interpretations of the site, by increasing the amount of arguments with which the in situ nature of the assemblage could be disproved (12).

The current situation places both Dikika and Lomekwi in epistemological limbo, but the consequences of both discoveries are major for human evolution. If true, most of our interpretations on the emergence of Homo, human behavior, stone tool use and encephalization should be discarded. For this reason, the correct interpretation of a) the Pliocene origin of Lomekwi-3 and, b) the hominin agency of the Dikika BSM are of crucial importance.

Here, 7 out of the 12 original marks from the two Dikika specimens were selected because the published images did not allow the use of the other marks. These 7 marks are represented by uneven quality images, which grant variable reliance to their classification. The virtual taphonomist shows this by lowering the probability threshold in those marks that exhibit the poorest quality. Results are discussed in the main text. It should be stressed that the most commonly identified mark class by the computer vision model was trampling. It should also be emphasized that one mark was classified as a cut mark.

BSM selected for analysis from (4):

DIK55-3D (modified from Supplementary Figure 16):

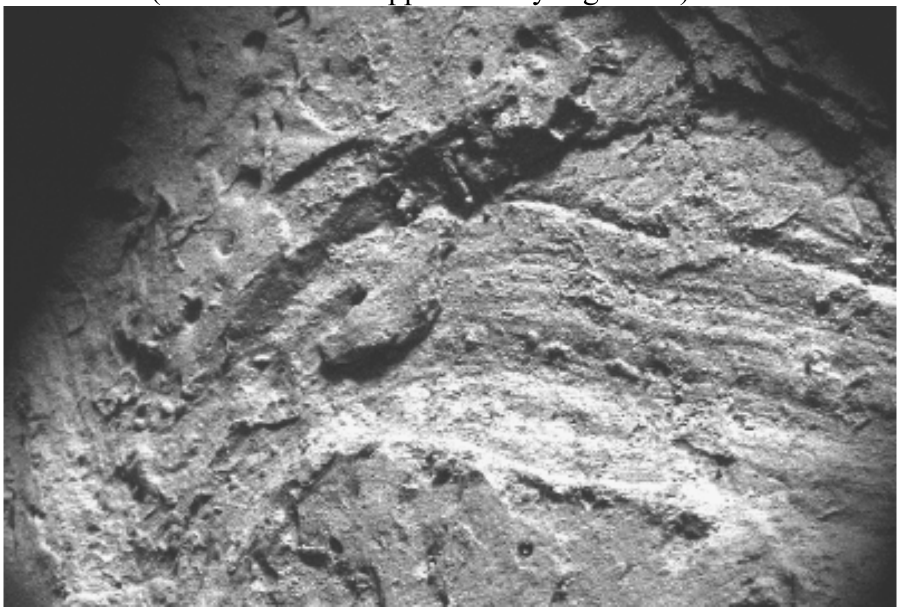

DIK55-3E (modified from Figure 3):

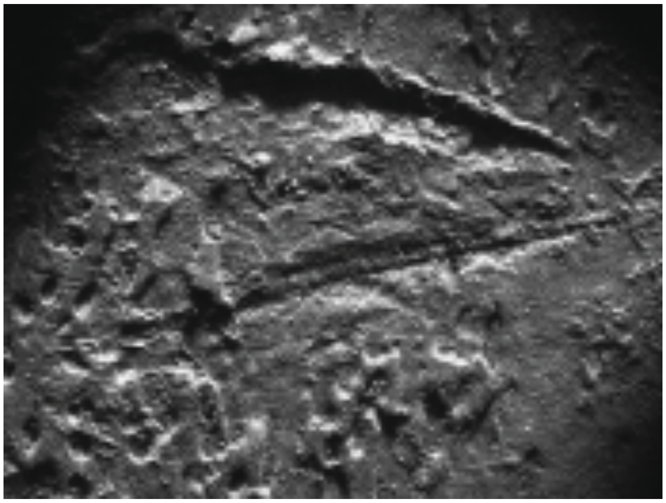

DIK55-3H2 (modified from Figure 3):

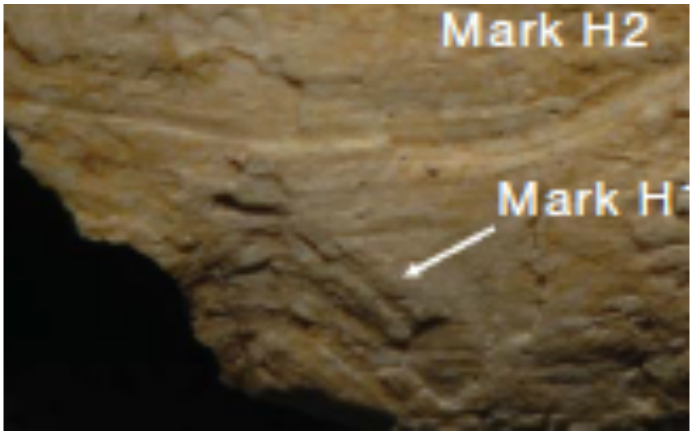

DIK55-3I (modified from Supplementary Figure 20):

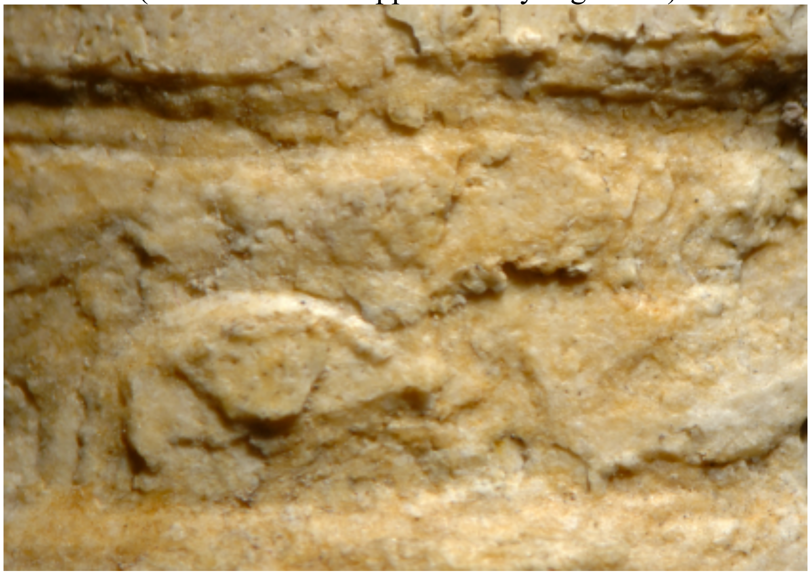

DIK55-3G1 (modified from Supplementary Figure 18):

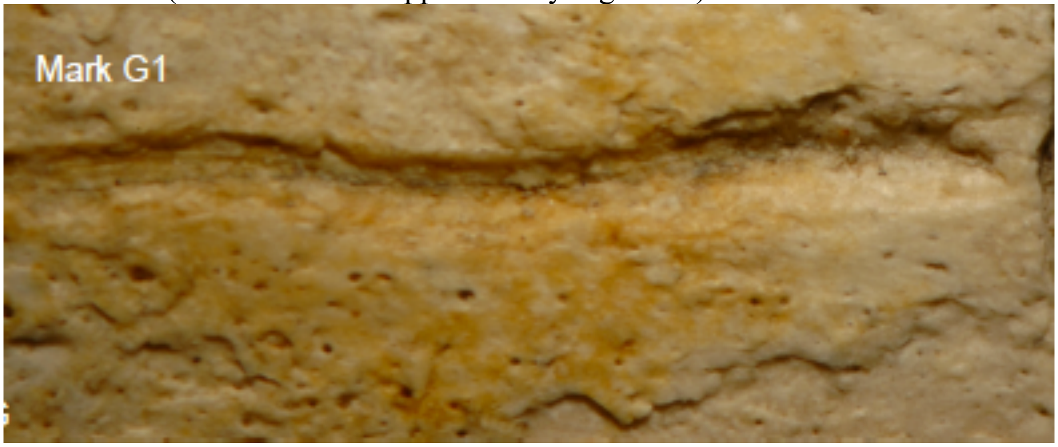

DIK55-2A2 (modified from Figure 3):

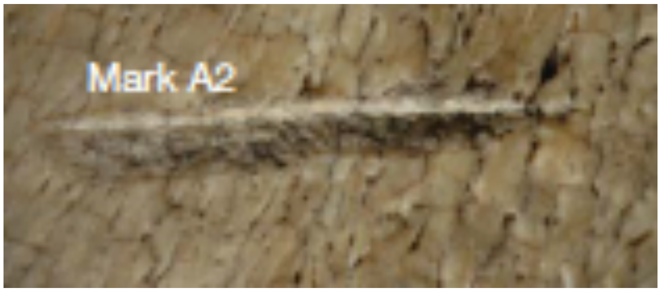

DIK55-2A1 and DIK55-2A2 (modified from Figure Supplementary 10):

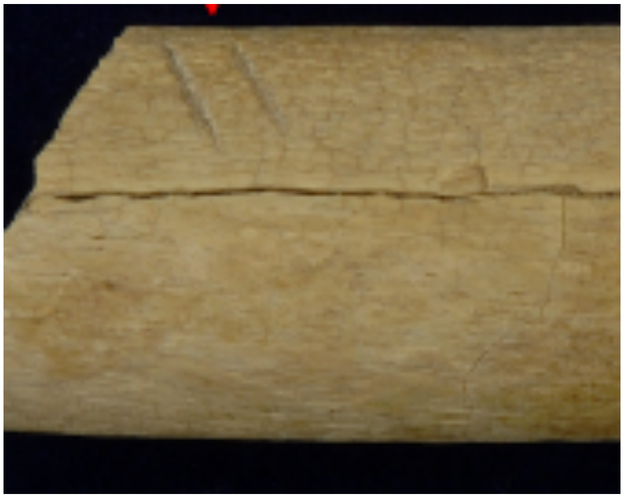

1c. Madagascar

In the past twenty years several claims have been made about a colonization of Madagascar prior to 1500 B.P. Cut-marked hippopotamus dated to 2000 B.P. (13) and cut-marked giant lemur bones dated to 2.700-2.200 B.P. (14) have been argued to indicate either an earlier presence of humans in Madagascar or support an initial colonization by African populations prior to the Austronesian arrival in the island. Some dates even extended further back in time to as much as 4000 B.P(15). A somewhat recent analysis of the extinct (Palaeopropithecus) and extant (Propithecus) lemur remains from Taolambiby (2700-2200 BP) has been presented as the most solid evidence of an old presence of humans on the island (14). However, lemur bones at the locality of Taolambiby have diverse provenience. Some Palaeopropithecus remains from this locality have yielded dates of 3000-1900 B.P (16). The older extinct Palaeopropithecus were succeeded by the extant Propithecus. Walker´s collection of Propithecus (>270 specimens) show abundant evidence of cut marks (9% of the sample). Most of the cutmarked bones in this collection have been dated to 1236+/- 900 B.P. (16). However, purported cut mark evidence has been reported for the older Palaeopropithecus remains (10 cutmarked specimens)(14). A recent review of this evidence argues that such purported human BSM are natural taphonomic marks caused by abrasion (16).

This recent revision on the issue of the earliest presence of humans on the island has been based on the largest collection of specimens taphonomically analyzed until present. The three main localities analyzed have been Ambolisatra (1300-1000 B.P.), Itampolo (1800-100 B.P.) and Taolambiby (3000-1000 B.P.) (16) have yielded a sample of 2710 specimens, representing 110 individuals, with megafuanla remains representing 77 MNI and 1787 NISP. The most abundant megafaunal remains are: hippopotamus, crocodile, giant tortoise, giant lemurs, elephant birds. This recent revision is skeptical than human presence in Madagascar could be justified beyond 1500 B.P. It is precisely on the elephant birds that the most recent controversy has arised. A recent work argues that ">10,500-year-old human-modified bones for the extinct elephant birds Aepyornis and Mullerornis, show perimortem chop marks, cut marks, and depression fractures consistent with immobilization and dismemberment". This purported evidence "for anthropogenic perimortem modification of directly dated bones represents the earliest indication of humans in Madagascar, predating all other archaeological and genetic evidence by >6000 years and changing our understanding of the history of human colonization of Madagascar." (17).

Here, we submitted images of one mark from a Hippopotamus femur from Anjohibe (dated between 2000 and 1400 B.P.) and another one from a Hippopotamus jugal bone from Itampolo (dated to 1800-1100 B.P.). The oldest purported cut mark analyzed is TajT-3 from the tibiotarsus of A. maximus (USNM A605209) from the Christmas River site (17). See results in the text.

Images selected for analysis:

ANU107-1 (Itampolo; jugal bone, Hippopotamus. Modified from (16)):

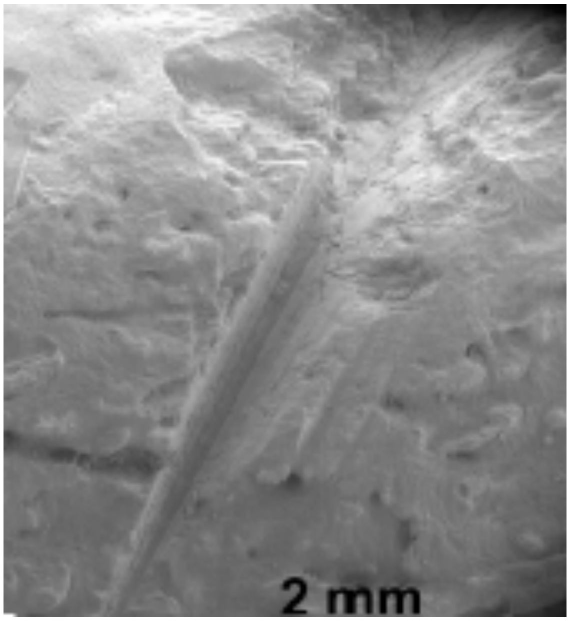

ANJ906´07 (Anjohibe: femur, Hippopotamus)(modified from (13), Figure 7):

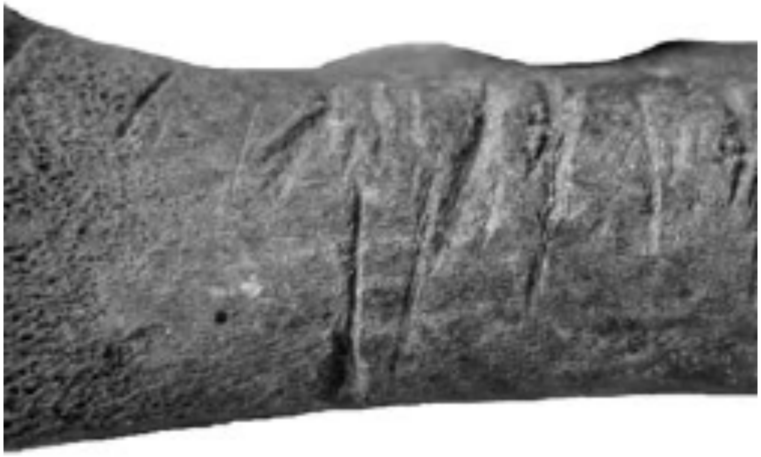

USNM A605209 (TT3) (Christmas River: distal tibiotarsus, A. maximus) (modified from (17), Figure 4c). Reprinted/adapted from [J. Hansford, et al., Early Holocene human presence in Madagascar evidenced by exploitation of avian megafauna. Sci Adv4, eaat6925 (2018)]. © The Authors, some rights reserved; exclusive licensee American Association for the Advancement of Science. Distributed under a Creative Commons Attribution NonCommercial License 4.0 (CC BY-NC) http://creativecommons.org/licenses/by-nc/4.0/:

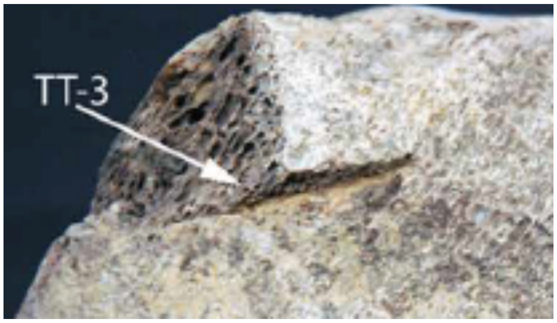

1d. Orce: the purported earliest cut marks in Europe

In the Guadix Baza region, a couple of sites (Barranco del León and Fuentenueva 3) have been used to defend a model of hominin kleptoparasitism from sabertooth felid kills and intense competition with other scavengers (18–22). These authors argue that modern studies with lions and leopards are irrelevant because in the early Pleistocene in the Iberian peninsula the main predators were only sabertooth felids (namely, *Megantereon* and *Homotherium*), which must have behaved very differently in their consumption of prey. The authors assume that these predators must have hunted larger ungulate prey relative to their body size and that they exploited their prey to a lesser extent, which would have resulted in greater amounts of flesh abandoned in the carcasses.

Both of these statements remain unsupported. Lions were predominant over sabertooth felids already in the African record from 2 Ma onwards. Given that the bulk of fauna accumulated at early archaeological sites in Africa were small and medium-sized taxa, this theoretically rules out sabertooth felids as potential sources of carrion for early hominins if these felids were focusing on larger game. Referential models made on modern felids are, thus, applicable to the African record without any concern. In the absence of competition, sabertooth felids in Eurasia during the same period may have interacted differently with their game. However, this needs to be proved rather than theoretically assumed. In the absence of competition from other felids, there is no support for inferring that those sabertooth felids unnecessarily engaged into hunting larger game when less risky and equally high-yielding strategies focusing on smaller game would have been available.

Modern lions do not need to systematically engage into hunting megafauna because they occupy a niche within the predatory guild that allows them to successfully obtain medium-sized carcasses without competition. However, when they reside in big prides in environments where megafauna abounds, lions can succeed in hunting not only juveniles but also adult elephant individuals (23–26). This evidence invalidates the first argument. Additionally, one should consider that modern predators target prey according to their caloric needs. No predator targets prey larger than what they need. When lions reside in low numbers or are solitary, they target smaller game, like topis or wildebeests or even gazelles for solitary males. When they live in larger prides, they target buffaloes (27). When they live in prides of dozens of individuals, they can attempt to bring down even elephants (Joubert, 2006). Even when looking at prehistoric predators prior to the emergence of the modern predatory guilds, carnivores show dentitions that

show their evolution to maximize exploitation of prey yields (28). Having a carnivore that kills prey of the size of an elephant or a rhinoceros for eating just a small amount of meat and abandoning carcasses almost complete is not only something unheard of in any known carnivore, but also poses the question of why targeting such large game when the purported limited need/capability of meat consumption by these sabertooth felids could also have been supplied and rendered easier if targeting much smaller game. It just does not make sense from an ecological point of view. Evolutionarily, it also posits problems. How such a wasteful strategy has been repeated several times given that sabertooths were subjected to frequent evolutionary convergence. Nimravidae and Barbourofelidae carnivore sabertooths converged so closely to Felidae sabertooths that natural selection must have been very restrictive for them (28, 29). These selective restrictions must have been so intense as to occur also among marsupials (e.g., Thylacosmilus) (30, 31), not to mention a diverse group of Permian reptiles, the therapsid gorgonopsians, which were the earliest true sabertooths. These were also followed by Eocene and Oligocene creodonts, displaying a diversity of sabertooth forms.

If one argues that the long canines would have prevented all these carnivores from efficient carcass defleshing, it would mean that a very wasteful strategy with no modern ecological counterpart would have been predominant over millions of years across the Neogene. We should remember that sabertooths were some of the predominant large carnivores in the Miocene along broad geographical areas and had they abandoned carcasses as complete as some researchers suggest, this would have prompted a wider array of carnivores in those ecosystems which would have carved a niche by facilitation enhanced by inefficient sabertooths. This is not documented in the paleontological record. It should also be emphasized that no study has convincingly shown that sabertooths could not use their dentition to efficiently deflesh their prey. Van Valkenburgh and Ruff (32) argued that some sabertooths avoided contact with the bone to avoid breaking the sabers. However, most sabertooths show heavy wear of carnassials, resulting from frequent bone contact, which shows that these felids teeth were efficiently used for intensive defleshing *(33).* Some gorgonopsians had all or most of their teeth anteriorly to the big sabers (e.g., Rubidgea), showing that those predators were also efficiently defleshing prey with a battery of teeth situated around the sabers.

Modern felids detach flesh from their prey with a lateral gripple of both carnassials. There is nothing in the biomechanics of sabertooth jaws to prevent them from having used the same strategy, despite the long sabers. It has even argued that the carnassials of several of these sabertooths were more efficient shearing and cutting than most modern carnivores (33). The longer carnassials of sabertooths compared to modern felids also show more intense wear than modern flesh-eating carnivores. This is not just an outcome of stronger force in bite, but also of intense use. Lever models to jaw mechanics show that sabertooths improved their leverage through a more posterior position of carnassials matching a more posterior position of chewing muscles. The leverage of the temporalis is similar to extant felids (33). The close distance of sabers to the lateral mandibular bodies restricted the mesio-lateral movement of jaws. This was solved by a rotation of both carnassials around the mesio-distal axis to permit occlusion. Therefore, no biomechanical model shows anything but efficient use of dentition for defleshing. This interpretation can be further supported by the more anterior position of the upper incisors in a more parabolic dental arcade in sabertooths compared to modern felids. This would have enabled sabertooths to use their incisors for defleshing more efficiently than modern felids. This

would explain why the incisors in this group of carnivores appear frequently more worn than in their modern felid counterparts.

For their second argument, these supporters of the idea that hominins scavenged from sabertooth kills have not provided any compelling evidence that sabertooth felids abandoned their prey in a more fleshed state than their modern counterparts (Rodríguez-Gómez et al., 2016; Espigares et al., 2019). In the absence of taphonomic evidence, this assumption remains highly speculative. The authors have assumed that these carnivores abandoned fleshed carcasses because their front dentition (especially their long canines) would have prevented them from defleshing efficiently their prey. However, these arguments stay in stark contradiction with both taphonomic and paleobiological evidence. The first type of evidence comes from the taphonomic study of Friesenhahn Cave (Texas) long interpreted as a *Homotherium* den, full of subadult proboscidean bones, where bones appear highly tooth marked, which is an indirect evidence of fairly complete carcass defleshing (34). The second type of evidence is the correlation found between wear on premolar dentition of carnivores and their frequency of contact with prey bone, which is an indirect proxy of intensity of defleshing during consumption (35, 36). Hartstone-Rose (2008: Evaluating the hominin scavenging niche through analysis of carcass-processing abilities of the carnivore guild) provided a thorough study in which he showed that all the iconic sabertooth taxa, but most specifically Homotherium and Megantereon had not only more contact with bone surfaces than modern lions, but their cusp wear was only similar to durophagous carnivores like hyenas, clearly indicating that they defleshed their prey more thoroughly than previously assumed. This interpretation derived from wear of dentition has recently been further supported by microwear patterns of the enamel. Dental Microwear Texture Analysis (MDTA) in Smilodon has shown that this predator did not avoid bone contact, being indistinguishable from modern lions, indirectly showing intense defleshing of their prey (37). A comparative MDTA on several Pleistocene carnivores including sabertooths shows that their microwear pattern of all of them was similar to modern carnivores and sabertooths included frequent bone breaking (38). The combination of these two types of evidence (sabertooth tooth wear and the taphonomy of Friesenhahn Cave), standing as the only empirical evidence of sabertooth modification of carcasses and state of carcasses upon abandonment, also rejects the second assumption made by researchers supporting that hominins were scavenging fairly complete carcasses from sabertooths. Therefore, cut marks showing intense butchery and defleshing by hominins in carcasses from small and medium-sized animals should indeed be the most tantalizing evidence for hominin primary access to them and, most likely, of hunting behavior as carcass acquisition strategy.

So, do these authors have anything empirically tangible beyond modeling and speculation for inferring that hominins were scavengers? Recently, they published a detailed account of the bone surface modifications that they found at Barranco del León and Fuentenueva 3. They found 32 cut marks on each site. Each site has multiple layers (two in Fuentenueva 3 and five in Barranco del León). Such a sample is too small to extract any meaningful interpretation. If spread over each level, this sample becomes even smaller. No statistical method can provide any significant support to any interpretation derived from such a split sample. Different behaviors and agencies over different levels may produce different cut mark patterns which would remain unappreciated by this scanty sample and whose interpretation would be biased if attributed solely to one particular depositional moment. Despite this, one could attempt to extract any information from

these limited cutmarked bone assemblages. There are referential models that would allow to interpret them as they are required following uniformitarian principles. Without attempting to link the cut mark data to any of these referents, (39) notice that most marks occur on long bone shafts from meating bones and interpret them as defleshing marks. This would be supported by the location of several cut marks on ribs, which are usually defleshed when accessing carcasses after felids. These authors also remark the presence of cut marks on the ventral side of vertebral bodies and rib suggestive of evisceration, which lead them to interpret that sometimes hominins had access to completely fleshed carcasses. This would contradict their previous interpretations of hominins having access to (what should be largely defleshed) carcasses from felid kills. If Megantereon was abandoning fleshed carcasses and exploiting only viscerae and meagre amounts of flesh, hominins should not have had a chance to eviscerate scavenged carcasses. These cut mark data also contradicts interpretations of opportunistic behaviors by hominins, especially when it has been argued by these authors that the landscape sustained intense competition between hominins and other scavengers (21, 40). This taphonomic evidence would also indirectly suggest early access to carcasses, which grants support to alternative interpretations of active predation of hominins on some of those carcasses. However, we stress again that sample size (especially if considered across all the levels from which it is derived) is too small to derive any meaningful interpretation.

However, all these poorly empirically-supported interpretations rest on the potentially controversial ground that bone surface modifications were correctly identified. We assume that the images provided by Espigares et al. (2019) are a selection of the best marks that their collection contains. Such an assumption may not be correct. A closer look at some of these marks promote doubts on their correct identification. For example, in our opinion, two of their four cut marks displayed cannot be confidently identified as such. One shows a set of very shallow and parallel oriented microstriations that are widely spaced in between and that are typical of the microabrasion generated during trampling (arrows in Fig 1A). No feature of that purported cut mark, other than its cross-section, invites any confidence in its attribution to a cut mark. Another mark is documented on a chemically-modified bone that preserved no clear feature that such a mark could be interpreted as a chop mark. As a matter of fact if it were a chop mark weathered by diagenesis, one would expect a single straight groove; instead, the groove looks as if it turns and continues in a different direction (Fig 1 B). Given the intensity of the weathering it is very difficult to ascribe any agency to such a mark. The presence of sedimentary abrasion is also documented in a third specimen where the most conspicuous marks (Fig 2, red arrows) are very different in length and width and would imply different effectors, not just one single flake edge; however, they would fit well a scenario of trampling by different particle sizes, as is suggested by the multiple microabrasion and microstriations that are found with random orientations along most of the specimen surface (Fig 2, yellow arrows). We would not feel confident in identifying some of the other marks that Espigares et al. (2019) display as carnivore marks as such either, because the bone specimen is leached and the marks highly weathered. With such a poor cortical preservation bone surface identification is highly risky and prone to error. Therefore, with a limited sample of poorly preserved marks, both Fuentenueva 3 and Barranco del León remain not well understood in terms of agency and process interaction. Any inferences made on hominin behavior beyond documenting some butchery in both assemblages will remain highly controversial and should not be taken seriously.

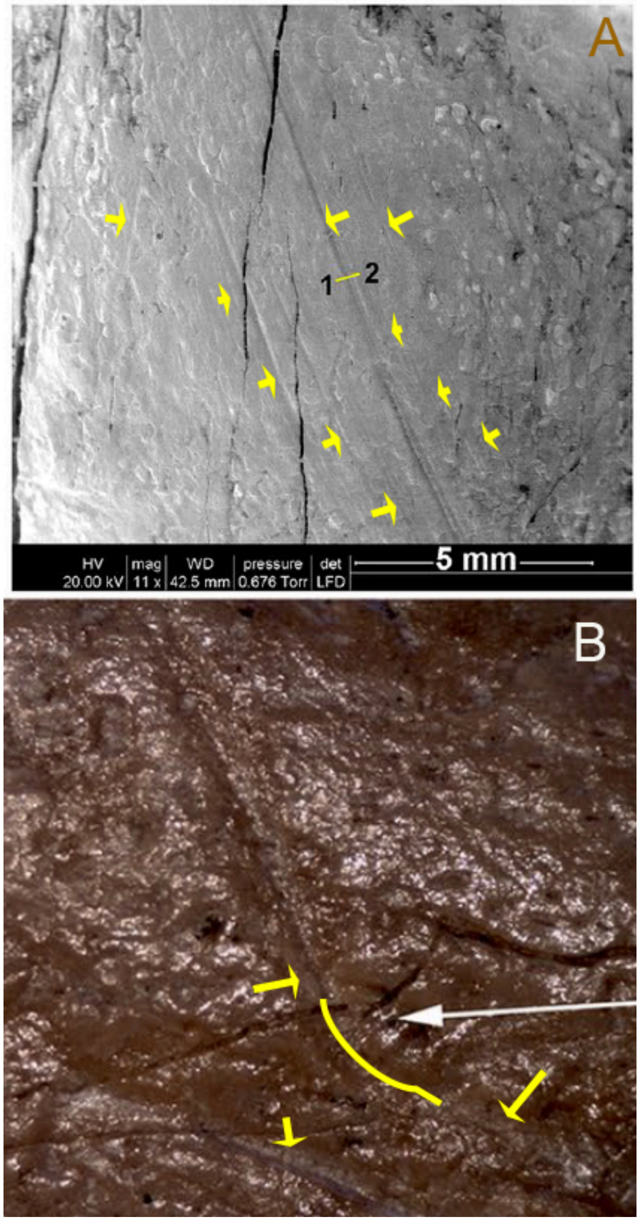

Figure 1. Two of the Fuentenueva BSM interpreted by (39) as cut marks (figures modified from the originals)

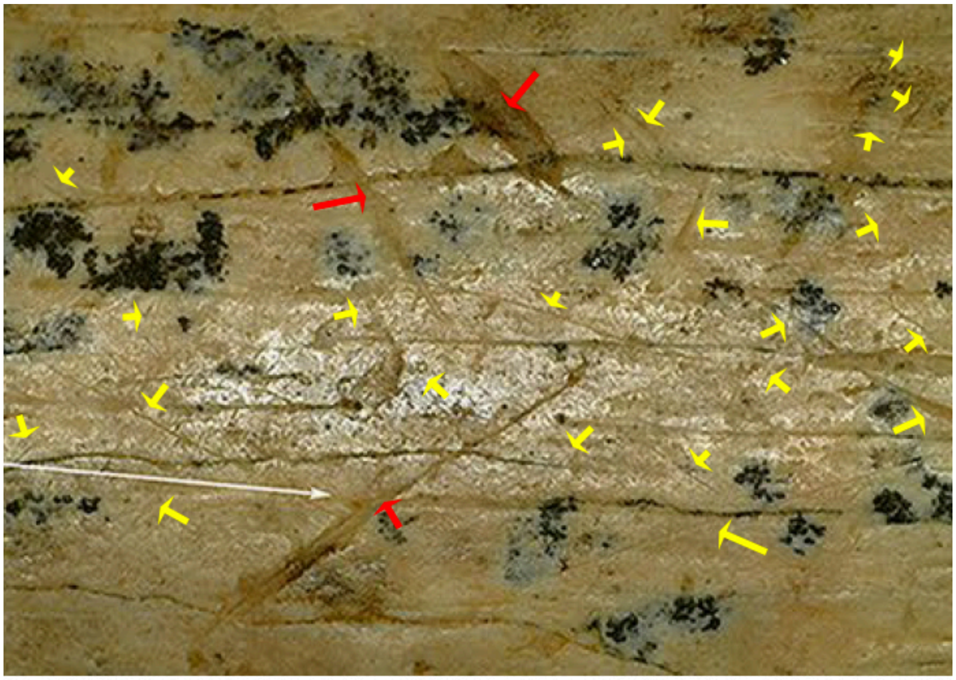

Figure 2. Some of the Fuentenueva BSM interpreted by (39) as cut marks and with conspicuous evidence as resulting from trampling. Modified from the original.

In the present study, we have only used two BSM because most of the images in the original publication are of such a poor quality that the intense pixelation made them highly unreliable for the DL analysis. The two images selected from (39) were:

FN3 (modified from the original) (Figure 3 b):

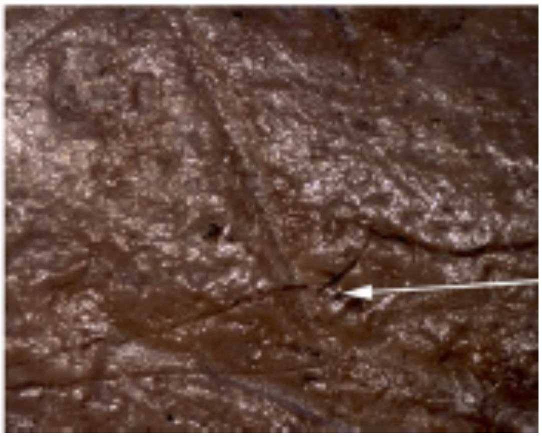

FN3 (modified from the original) (Figure 3 c):

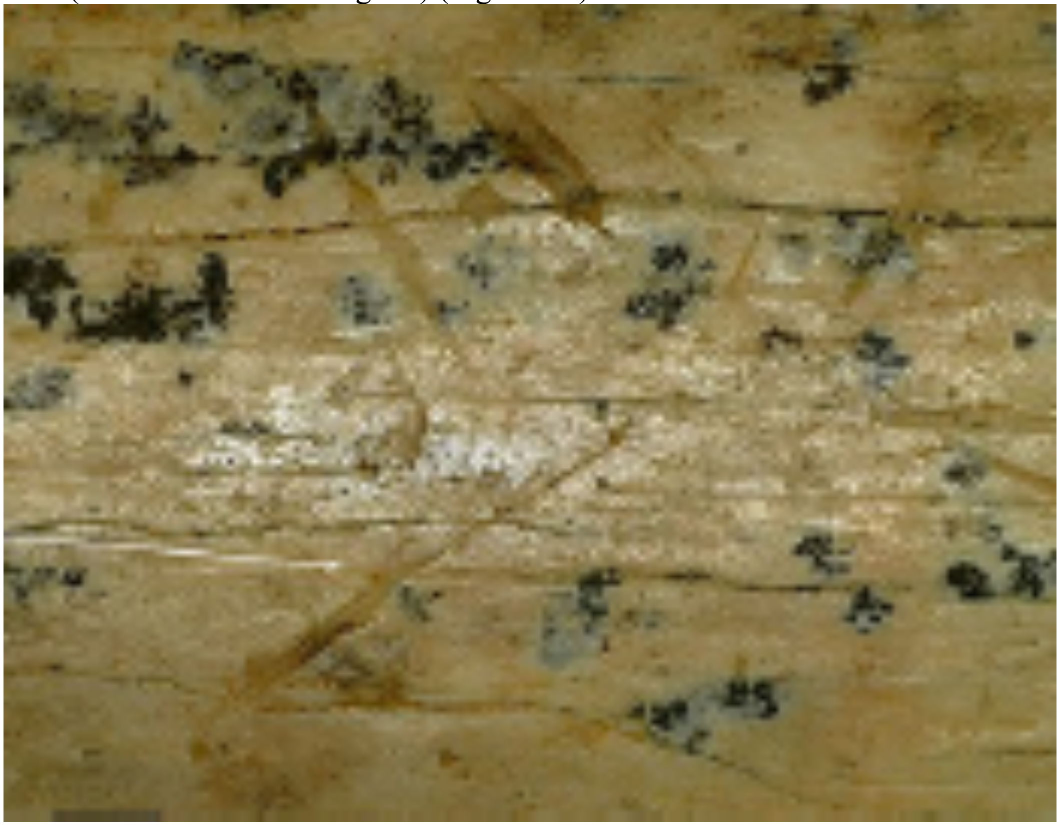

2. Model architectural parameters

Table S1. Model architecture for the Alexnet model

Layer (type)	Output Shape	Parameter #
conv2d_11 (Conv2D)	(None, 18, 98, 96)	23424
activation_19 (Activation)	(None, 18, 98, 96)	0
max_pooling2d_7 (MaxPooling2	(None, 9, 49, 96)	0
batch_normalization_17 (Batc	(None, 9, 49, 96)	384
conv2d_12 (Conv2D)	(None, 1, 41, 256)	1990912
activation_20 (Activation)	(None, 1, 41, 256)	0
max_pooling2d_8 (MaxPooling2	(None, 1, 21, 256)	0
batch_normalization_18 (Batc	(None, 1, 21, 256)	1024
conv2d_13 (Conv2D)	(None, 1, 21, 384)	98688
activation_21 (Activation)	(None, 1, 21, 384)	0
batch_normalization_19 (Batc	(None, 1, 21, 384)	1536
conv2d_14 (Conv2D)	(None, 1, 21, 384)	147840
activation_22 (Activation)	(None, 1, 21, 384)	0
batch_normalization_20 (Batc	(None, 1, 21, 384)	1536
conv2d_15 (Conv2D)	(None, 1, 21, 256)	98560
activation_23 (Activation)	(None, 1, 21, 256)	0
max_pooling2d_9 (MaxPooling2	(None, 1, 11, 256)	0
batch_normalization_21 (Batc	(None, 1, 11, 256)	1024
flatten_3 (Flatten)	(None, 2816)	0
dense_9 (Dense)	(None, 4096)	11538432
activation_24 (Activation)	(None, 4096)	0
dropout_7 (Dropout)	(None, 4096)	0
batch_normalization_22 (Batc	(None, 4096)	16384
dense_10 (Dense)	(None, 4096)	16781312
activation_25 (Activation)	(None, 4096)	0
dropout_8 (Dropout)	(None, 4096)	0
batch_normalization_23 (Batc	(None, 4096)	16384
dense_11 (Dense)	(None, 1000)	4097000
activation_26 (Activation)	(None, 1000)	0
dropout_9 (Dropout)	(None, 1000)	0
dropout_9 (Dropout)	(None, 1000)	0
batch_normalization_24 (Batc	(None, 1000)	4000
dense_12 (Dense)	(None, 1)	1001
activation_27 (Activation)	(None, 1)	0
Total params: 34,819,441<br>Trainable params: 34,798,305<br>Non-trainable params: 21,136		

Table S2. Model architecture for the Jason 1 model

Layer (type)	Output Shape	Param #
conv2d_1 (Conv2D)	(None, 78, 398, 32)	896
max_pooling2d_1 (MaxPooling2	(None, 39, 199, 32)	0
conv2d_2 (Conv2D)	(None, 37, 197, 64)	18496
max_pooling2d_2 (MaxPooling2	(None, 18, 98, 64)	0
conv2d_3 (Conv2D)	(None, 16, 96, 128)	73856
max_pooling2d_3 (MaxPooling2	(None, 8, 48, 128)	0
conv2d_4 (Conv2D)	(None, 6, 46, 128)	147584
max_pooling2d_4 (MaxPooling2	(None, 3, 23, 128)	0
flatten_1 (Flatten)	(None, 8832)	0
dense_1 (Dense)	(None, 512)	4522496
dense_2 (Dense)	(None, 3)	1539
Total params: 4,764,867<br>Trainable params: 4,764,867<br>Non-trainable params: 0		

Table S3. Model architecture for the Jason 2 model.

**Layer (type)**	**Output Shape**	**Param #**
conv2d_40 (Conv2D)	(None, 40, 200, 32)	7808
batch_normalization_45 (Batc	(None, 40, 200, 32)	128
conv2d_41 (Conv2D)	(None, 20, 100, 32)	82976
batch_normalization_46 (Batc	(None, 20, 100, 32)	128
max_pooling2d_21 (MaxPooling	(None, 20, 100, 32)	0
dropout_19 (Dropout)	(None, 20, 100, 32)	0
conv2d_42 (Conv2D)	(None, 10, 50, 64)	165952
batch_normalization_47 (Batc	(None, 10, 50, 64)	256
conv2d_43 (Conv2D)	(None, 5, 25, 64)	331840
batch_normalization_48 (Batc	(None, 5, 25, 64)	256
max_pooling2d_22 (MaxPooling	(None, 5, 25, 64)	0
dropout_20 (Dropout)	(None, 5, 25, 64)	0
conv2d_44 (Conv2D)	(None, 3, 13, 128)	663680
batch_normalization_49 (Batc	(None, 3, 13, 128)	512
conv2d_45 (Conv2D)	(None, 2, 7, 128)	1327232
batch_normalization_50 (Batc	(None, 2, 7, 128)	512
max_pooling2d_23 (MaxPooling	(None, 2, 7, 128)	0
dropout_21 (Dropout)	(None, 2, 7, 128)	0
conv2d_46 (Conv2D)	(None, 1, 4, 512)	5308928
batch_normalization_51 (Batc	(None, 1, 4, 512)	2048
conv2d_47 (Conv2D)	(None, 1, 2, 512)	21234176
batch_normalization_52 (Batc	(None, 1, 2, 512)	2048
max_pooling2d_24 (MaxPooling	(None, 1, 2, 512)	0
dropout_22 (Dropout)	(None, 1, 2, 512)	0
flatten_4 (Flatten)	(None, 1024)	0
dense_9 (Dense)	(None, 128)	131200
batch_normalization_53 (Batc	(None, 128)	512
dropout_23 (Dropout)	(None, 128)	0
dense_10 (Dense)	(None, 1)	129
Total params: 29,260,321<br>Trainable params: 29,257,121		

Non-trainable params: 3,200		

Table S4. Model architecture for the VGG16 model.

Layer (type)	Output Shape	Param #
input_1 (InputLayer)	(None, 80, 400, 3)	0
block1_conv1 (Conv2D)	(None, 80, 400, 64)	1792
block1_conv2 (Conv2D)	(None, 80, 400, 64)	36928
block1_pool (MaxPooling2D)	(None, 40, 200, 64)	0
block2_conv1 (Conv2D)	(None, 40, 200, 128)	73856
block2_conv2 (Conv2D)	(None, 40, 200, 128)	147584
block2_pool (MaxPooling2D)	(None, 20, 100, 128)	0
block3_conv1 (Conv2D)	(None, 20, 100, 256)	295168
block3_conv2 (Conv2D)	(None, 20, 100, 256)	590080
block3_conv3 (Conv2D)	(None, 20, 100, 256)	590080
block3_pool (MaxPooling2D)	(None, 10, 50, 256)	0
block4_conv1 (Conv2D)	(None, 10, 50, 512)	1180160
block4_conv2 (Conv2D)	(None, 10, 50, 512)	2359808
block4_conv3 (Conv2D)	(None, 10, 50, 512)	2359808
block4_pool (MaxPooling2D)	(None, 5, 25, 512)	0
block5_conv1 (Conv2D)	(None, 5, 25, 512)	2359808
block5_conv2 (Conv2D)	(None, 5, 25, 512)	2359808
block5_conv3 (Conv2D)	(None, 5, 25, 512)	2359808
block5_pool (MaxPooling2D)	(None, 2, 12, 512)	0
flatten_2 (Flatten)	(None, 12288)	0
dense_3 (Dense)	(None, 128)	1572992
dense_4 (Dense)	(None, 3)	387
Total params: 16,288,067<br>Trainable params: 1,573,379<br>Non-trainable params: 14,714,688		

Table S5. Model architecture for the Resnet50 model.

Layer (type)	Output Shape	Param #	Connected to
input_2 (InputLayer)	(None, 80, 400, 3)	0	
conv1_pad (ZeroPadding2D)	(None, 86, 406, 3)	0	input_2[0][0]
conv1 (Conv2D)	(None, 40, 200, 64)	9472	conv1_pad[0][0]
bn_conv1 (BatchNormalization)	(None, 40, 200, 64)	256	conv1[0][0]
activation_1 (Activation)	(None, 40, 200, 64)	0	bn_conv1[0][0]
pool1_pad (ZeroPadding2D)	(None, 42, 202, 64)	0	activation_1[0][0]
max_pooling2d_5 (MaxPooling2D)	(None, 20, 100, 64)	0	pool1_pad[0][0]
res2a_branch2a (Conv2D)	(None, 20, 100, 64)	4160	max_pooling2d_5[0][0]
bn2a_branch2a (BatchNormalizati	(None, 20, 100, 64)	256	res2a_branch2a[0][0]
activation_2 (Activation)	(None, 20, 100, 64)	0	bn2a_branch2a[0][0]
res2a_branch2b (Conv2D)	(None, 20, 100, 64)	36928	activation_2[0][0]
bn2a_branch2b (BatchNormalizati	(None, 20, 100, 64)	256	res2a_branch2b[0][0]
activation_3 (Activation)	(None, 20, 100, 64)	0	bn2a_branch2b[0][0]
res2a_branch2c (Conv2D)	(None, 20, 100, 256)	16640	activation_3[0][0]
res2a_branch1 (Conv2D)	(None, 20, 100, 256)	16640	max_pooling2d_5[0][0]
bn2a_branch2c (BatchNormalizati	(None, 20, 100, 256)	1024	res2a_branch2c[0][0]
bn2a_branch1 (BatchNormalizatio	(None, 20, 100, 256)	1024	res2a_branch1[0][0]
add_1 (Add)	(None, 20, 100, 256)	0	bn2a_branch2c[0][0]<br>bn2a_branch1[0][0]
activation_4 (Activation)	(None, 20, 100, 256)	0	add_1[0][0]
res2b_branch2a (Conv2D)	(None, 20, 100, 64)	16448	activation_4[0][0]
bn2b_branch2a (BatchNormalizati	(None, 20, 100, 64)	256	res2b_branch2a[0][0]
activation_5 (Activation)	(None, 20, 100, 64)	0	bn2b_branch2a[0][0]
res2b_branch2b (Conv2D)	(None, 20, 100, 64)	36928	activation_5[0][0]
bn2b_branch2b (BatchNormalizati	(None, 20, 100, 64)	256	res2b_branch2b[0][0]
activation_6 (Activation)	(None, 20, 100, 64)	0	bn2b_branch2b[0][0]
res2b_branch2c (Conv2D)	(None, 20, 100, 256)	16640	activation_6[0][0]
bn2b_branch2c (BatchNormalizati	(None, 20, 100, 256)	1024	res2b_branch2c[0][0]
add_2 (Add)	(None, 20, 100, 256)	0	bn2b_branch2c[0][0]<br>activation_4[0][0]
activation_7 (Activation)	(None, 20, 100, 256)	0	add_2[0][0]
res2c_branch2a (Conv2D)	(None, 20, 100, 64)	16448	activation_7[0][0]
bn2c_branch2a (BatchNormalizati	(None, 20, 100, 64)	256	res2c_branch2a[0][0]
activation_8 (Activation)	(None, 20, 100, 64)	0	bn2c_branch2a[0][0]
res2c_branch2b (Conv2D)	(None, 20, 100, 64)	36928	activation_8[0][0]
bn2c_branch2b (BatchNormalizati	(None, 20, 100, 64)	256	res2c_branch2b[0][0]
activation_9 (Activation)	(None, 20, 100, 64)	0	bn2c_branch2b[0][0]
res2c_branch2c (Conv2D)	(None, 20, 100, 256)	16640	activation_9[0][0]
bn2c_branch2c (BatchNormalizati	(None, 20, 100, 256)	1024	res2c_branch2c[0][0]
add_3 (Add)	(None, 20, 100, 256)	0	bn2c_branch2c[0][0]<br>activation_7[0][0]
activation_10 (Activation)	(None, 20, 100, 256)	0	add_3[0][0]
res3a_branch2a (Conv2D)	(None, 10, 50, 128)	32896	activation_10[0][0]
bn3a_branch2a (BatchNormalizati	(None, 10, 50, 128)	512	res3a_branch2a[0][0]

activation_11 (Activation)	(None, 10, 50, 128)	0	bn3a_branch2a[0][0]
res3a_branch2b (Conv2D)	(None, 10, 50, 128)	147584	activation_11[0][0]
bn3a_branch2b (BatchNormalizati	(None, 10, 50, 128)	512	res3a_branch2b[0][0]
activation_12 (Activation)	(None, 10, 50, 128)	0	bn3a_branch2b[0][0]
res3a_branch2c (Conv2D)	(None, 10, 50, 512)	66048	activation_12[0][0]
res3a_branch1 (Conv2D)	(None, 10, 50, 512)	131584	activation_10[0][0]
bn3a_branch2c (BatchNormalizati	(None, 10, 50, 512)	2048	res3a_branch2c[0][0]
bn3a_branch1 (BatchNormalizatio	(None, 10, 50, 512)	2048	res3a_branch1[0][0]
add_4 (Add)	(None, 10, 50, 512)	0	bn3a_branch2c[0][0]<br>bn3a_branch1[0][0]
activation_13 (Activation)	(None, 10, 50, 512)	0	add_4[0][0]
res3b_branch2a (Conv2D)	(None, 10, 50, 128)	65664	activation_13[0][0]
bn3b_branch2a (BatchNormalizati	(None, 10, 50, 128)	512	res3b_branch2a[0][0]
activation_14 (Activation)	(None, 10, 50, 128)	0	bn3b_branch2a[0][0]
res3b_branch2b (Conv2D)	(None, 10, 50, 128)	147584	activation_14[0][0]
bn3b_branch2b (BatchNormalizati	(None, 10, 50, 128)	512	res3b_branch2b[0][0]
activation_15 (Activation)	(None, 10, 50, 128)	0	bn3b_branch2b[0][0]
res3b_branch2c (Conv2D)	(None, 10, 50, 512)	66048	activation_15[0][0]
bn3b_branch2c (BatchNormalizati	(None, 10, 50, 512)	2048	res3b_branch2c[0][0]
add_5 (Add)	(None, 10, 50, 512)	0	bn3b_branch2c[0][0]<br>activation_13[0][0]
activation_16 (Activation)	(None, 10, 50, 512)	0	add_5[0][0]
res3c_branch2a (Conv2D)	(None, 10, 50, 128)	65664	activation_16[0][0]
bn3c_branch2a (BatchNormalizati	(None, 10, 50, 128)	512	res3c_branch2a[0][0]
activation_17 (Activation)	(None, 10, 50, 128)	0	bn3c_branch2a[0][0]
res3c_branch2b (Conv2D)	(None, 10, 50, 128)	147584	activation_17[0][0]
bn3c_branch2b (BatchNormalizati	(None, 10, 50, 128)	512	res3c_branch2b[0][0]
activation_18 (Activation)	(None, 10, 50, 128)	0	bn3c_branch2b[0][0]
res3c_branch2c (Conv2D)	(None, 10, 50, 512)	66048	activation_18[0][0]
bn3c_branch2c (BatchNormalizati	(None, 10, 50, 512)	2048	res3c_branch2c[0][0]
add_6 (Add)	(None, 10, 50, 512)	0	bn3c_branch2c[0][0]<br>activation_16[0][0]
activation_19 (Activation)	(None, 10, 50, 512)	0	add_6[0][0]
res3d_branch2a (Conv2D)	(None, 10, 50, 128)	65664	activation_19[0][0]
bn3d_branch2a (BatchNormalizati	(None, 10, 50, 128)	512	res3d_branch2a[0][0]
activation_20 (Activation)	(None, 10, 50, 128)	0	bn3d_branch2a[0][0]
res3d_branch2b (Conv2D)	(None, 10, 50, 128)	147584	activation_20[0][0]
bn3d_branch2b (BatchNormalizati	(None, 10, 50, 128)	512	res3d_branch2b[0][0]
activation_21 (Activation)	(None, 10, 50, 128)	0	bn3d_branch2b[0][0]
res3d_branch2c (Conv2D)	(None, 10, 50, 512)	66048	activation_21[0][0]
bn3d_branch2c (BatchNormalizati	(None, 10, 50, 512)	2048	res3d_branch2c[0][0]
add_7 (Add)	(None, 10, 50, 512)	0	bn3d_branch2c[0][0]<br>activation_19[0][0]

activation_22 (Activation)	(None, 10, 50, 512)	0	add_7[0][0]
res4a_branch2a (Conv2D)	(None, 5, 25, 256)	131328	activation_22[0][0]
bn4a_branch2a (BatchNormalizati	(None, 5, 25, 256)	1024	res4a_branch2a[0][0]
activation_23 (Activation)	(None, 5, 25, 256)	0	bn4a_branch2a[0][0]
res4a_branch2b (Conv2D)	(None, 5, 25, 256)	590080	activation_23[0][0]
bn4a_branch2b (BatchNormalizati	(None, 5, 25, 256)	1024	res4a_branch2b[0][0]
activation_24 (Activation)	(None, 5, 25, 256)	0	bn4a_branch2b[0][0]
res4a_branch2c (Conv2D)	(None, 5, 25, 1024)	263168	activation_24[0][0]
res4a_branch1 (Conv2D)	(None, 5, 25, 1024)	52312	activation_22[0][0]
bn4a_branch2c (BatchNormalizati	(None, 5, 25, 1024)	4096	res4a_branch2c[0][0]
bn4a_branch1 (BatchNormalizatio	(None, 5, 25, 1024)	4096	res4a_branch1[0][0]
add_8 (Add)	(None, 5, 25, 1024)	0	bn4a_branch2c[0][0]<br>bn4a_branch1[0][0]
activation_25 (Activation)	(None, 5, 25, 1024)	0	add_8[0][0]
res4b_branch2a (Conv2D)	(None, 5, 25, 256)	262400	activation_25[0][0]
bn4b_branch2a (BatchNormalizati	(None, 5, 25, 256)	1024	res4b_branch2a[0][0]
activation_26 (Activation)	(None, 5, 25, 256)	0	bn4b_branch2a[0][0]
res4b_branch2b (Conv2D)	(None, 5, 25, 256)	590080	activation_26[0][0]
bn4b_branch2b (BatchNormalizati	(None, 5, 25, 256)	1024	res4b_branch2b[0][0]
activation_27 (Activation)	(None, 5, 25, 256)	0	bn4b_branch2b[0][0]
res4b_branch2c (Conv2D)	(None, 5, 25, 1024)	263168	activation_27[0][0]
bn4b_branch2c (BatchNormalizati	(None, 5, 25, 1024)	4096	res4b_branch2c[0][0]
add_9 (Add)	(None, 5, 25, 1024)	0	bn4b_branch2c[0][0]<br>activation_25[0][0]
activation_28 (Activation)	(None, 5, 25, 1024)	0	add_9[0][0]
res4c_branch2a (Conv2D)	(None, 5, 25, 256)	262400	activation_28[0][0]
bn4c_branch2a (BatchNormalizati	(None, 5, 25, 256)	1024	res4c_branch2a[0][0]
activation_29 (Activation)	(None, 5, 25, 256)	0	bn4c_branch2a[0][0]
res4c_branch2b (Conv2D)	(None, 5, 25, 256)	590080	activation_29[0][0]
bn4c_branch2b (BatchNormalizati	(None, 5, 25, 256)	1024	res4c_branch2b[0][0]
activation_30 (Activation)	(None, 5, 25, 256)	0	bn4c_branch2b[0][0]
res4c_branch2c (Conv2D)	(None, 5, 25, 1024)	263168	activation_30[0][0]
bn4c_branch2c (BatchNormalizati	(None, 5, 25, 1024)	4096	res4c_branch2c[0][0]
add_10 (Add)	(None, 5, 25, 1024)	0	bn4c_branch2c[0][0]<br>activation_28[0][0]
activation_31 (Activation)	(None, 5, 25, 1024)	0	add_10[0][0]
res4d_branch2a (Conv2D)	(None, 5, 25, 256)	262400	activation_31[0][0]
bn4d_branch2a (BatchNormalizati	(None, 5, 25, 256)	1024	res4d_branch2a[0][0]
activation_32 (Activation)	(None, 5, 25, 256)	0	bn4d_branch2a[0][0]
res4d_branch2b (Conv2D)	(None, 5, 25, 256)	590080	activation_32[0][0]
bn4d_branch2b (BatchNormalizati	(None, 5, 25, 256)	1024	res4d_branch2b[0][0]
activation_33 (Activation)	(None, 5, 25, 256)	0	bn4d_branch2b[0][0]
res4d_branch2c (Conv2D)	(None, 5, 25, 1024)	263168	activation_33[0][0]

bn4d_branch2c (BatchNormalizati	(None, 5, 25, 1024)	4096	res4d_branch2c[0][0]
add_11 (Add)	(None, 5, 25, 1024)	0	bn4d_branch2c[0][0] activation_31[0][0]
activation_34 (Activation)	(None, 5, 25, 1024)	0	add_11[0][0]
res4e_branch2a (Conv2D)	(None, 5, 25, 256)	262400	activation_34[0][0]
bn4e_branch2a (BatchNormalizati	(None, 5, 25, 256)	1024	res4e_branch2a[0][0]
activation_35 (Activation)	(None, 5, 25, 256)	0	bn4e_branch2a[0][0]
res4e_branch2b (Conv2D)	(None, 5, 25, 256)	590080	activation_35[0][0]
bn4e_branch2b (BatchNormalizati	(None, 5, 25, 256)	1024	res4e_branch2b[0][0]
activation_36 (Activation)	(None, 5, 25, 256)	0	bn4e_branch2b[0][0]
res4e_branch2c (Conv2D)	(None, 5, 25, 1024)	263168	activation_36[0][0]
bn4e_branch2c (BatchNormalizati	(None, 5, 25, 1024)	4096	res4e_branch2c[0][0]
add_12 (Add)	(None, 5, 25, 1024)	0	bn4e_branch2c[0][0] activation_34[0][0]
activation_37 (Activation)	(None, 5, 25, 1024)	0	add_12[0][0]
res4f_branch2a (Conv2D)	(None, 5, 25, 256)	262400	activation_37[0][0]
bn4f_branch2a (BatchNormalizati	(None, 5, 25, 256)	1024	res4f_branch2a[0][0]
activation_38 (Activation)	(None, 5, 25, 256)	0	bn4f_branch2a[0][0]
res4f_branch2b (Conv2D)	(None, 5, 25, 256)	590080	activation_38[0][0]
bn4f_branch2b (BatchNormalizati	(None, 5, 25, 256)	1024	res4f_branch2b[0][0]
activation_39 (Activation)	(None, 5, 25, 256)	0	bn4f_branch2b[0][0]
res4f_branch2c (Conv2D)	(None, 5, 25, 1024)	263168	activation_39[0][0]
bn4f_branch2c (BatchNormalizati	(None, 5, 25, 1024)	4096	res4f_branch2c[0][0]
add_13 (Add)	(None, 5, 25, 1024)	0	bn4f_branch2c[0][0] activation_37[0][0]
activation_40 (Activation)	(None, 5, 25, 1024)	0	add_13[0][0]
res5a_branch2a (Conv2D)	(None, 3, 13, 512)	524800	activation_40[0][0]
bn5a_branch2a (BatchNormalizati	(None, 3, 13, 512)	2048	res5a_branch2a[0][0]
activation_41 (Activation)	(None, 3, 13, 512)	0	bn5a_branch2a[0][0]
res5a_branch2b (Conv2D)	(None, 3, 13, 512)	2359808	activation_41[0][0]
bn5a_branch2b (BatchNormalizati	(None, 3, 13, 512)	2048	res5a_branch2b[0][0]
activation_42 (Activation)	(None, 3, 13, 512)	0	bn5a_branch2b[0][0]
res5a_branch2c (Conv2D)	(None, 3, 13, 2048)	1050624	activation_42[0][0]
res5a_branch1 (Conv2D)	(None, 3, 13, 2048)	2099200	activation_40[0][0]
bn5a_branch2c (BatchNormalizati	(None, 3, 13, 2048)	8192	res5a_branch2c[0][0]
bn5a_branch1 (BatchNormalizatio	(None, 3, 13, 2048)	8192	res5a_branch1[0][0]
add_14 (Add)	(None, 3, 13, 2048)	0	bn5a_branch2c[0][0] bn5a_branch1[0][0]
activation_43 (Activation)	(None, 3, 13, 2048)	0	add_14[0][0]
res5b_branch2a (Conv2D)	(None, 3, 13, 512)	1049088	activation_43[0][0]
bn5b_branch2a (BatchNormalizati	(None, 3, 13, 512)	2048	res5b_branch2a[0][0]
activation_44 (Activation)	(None, 3, 13, 512)	0	bn5b_branch2a[0][0]
res5b_branch2b (Conv2D)	(None, 3, 13, 512)	2359808	activation_44[0][0]
bn5b_branch2b (BatchNormalizati	(None, 3, 13, 512)	2048	res5b_branch2b[0][0]

activation_45 (Activation)	(None, 3, 13, 512)	0	bn5b_branch2b[0][0]
res5b_branch2c (Conv2D)	(None, 3, 13, 2048)	1050624	activation_45[0][0]
bn5b_branch2c (BatchNormalizati	(None, 3, 13, 2048)	8192	res5b_branch2c[0][0]
add_15 (Add)	(None, 3, 13, 2048)	0	bn5b_branch2c[0][0]<br>activation_43[0][0]
activation_46 (Activation)	(None, 3, 13, 2048)	0	add_15[0][0]
res5c_branch2a (Conv2D)	(None, 3, 13, 512)	1049088	activation_46[0][0]
bn5c_branch2a (BatchNormalizati	(None, 3, 13, 512)	2048	res5c_branch2a[0][0]
activation_47 (Activation)	(None, 3, 13, 512)	0	bn5c_branch2a[0][0]
res5c_branch2b (Conv2D)	(None, 3, 13, 512)	2359808	activation_47[0][0]
bn5c_branch2b (BatchNormalizati	(None, 3, 13, 512)	2048	res5c_branch2b[0][0]
activation_48 (Activation)	(None, 3, 13, 512)	0	bn5c_branch2b[0][0]
res5c_branch2c (Conv2D)	(None, 3, 13, 2048)	1050624	activation_48[0][0]
bn5c_branch2c (BatchNormalizati	(None, 3, 13, 2048)	8192	res5c_branch2c[0][0]
add_16 (Add)	(None, 3, 13, 2048)	0	bn5c_branch2c[0][0]<br>activation_46[0][0]
activation_49 (Activation)	(None, 3, 13, 2048)	0	add_16[0][0]
flatten_3 (Flatten)	(None, 79872)	0	activation_49[0][0]
dense_5 (Dense)	(None, 128)	10223744	flatten_3[0][0]
dense_6 (Dense)	(None, 3)	387	dense_5[0][0]
Total params: 33,811,843<br>Trainable params: 10,224,131<br>Non-trainable params: 23,587,712			

Table S6. Model architecture for the InceptionV3 model.

Layer (type)	Output Shape	Param #	Connected to
input_3 (InputLayer)	(None, 80, 400, 3)	0	
conv2d_5 (Conv2D)	(None, 39, 199, 32)	864	input_3[0][0]
batch_normalization_1 (BatchNor	(None, 39, 199, 32)	96	conv2d_5[0][0]
activation_50 (Activation)	(None, 39, 199, 32)	0	batch_normalization_1[0][0]
conv2d_6 (Conv2D)	(None, 37, 197, 32)	9216	activation_50[0][0]
batch_normalization_2 (BatchNor	(None, 37, 197, 32)	96	conv2d_6[0][0]
activation_51 (Activation)	(None, 37, 197, 32)	0	batch_normalization_2[0][0]
conv2d_7 (Conv2D)	(None, 37, 197, 64)	18432	activation_51[0][0]
batch_normalization_3 (BatchNor	(None, 37, 197, 64)	192	conv2d_7[0][0]
activation_52 (Activation)	(None, 37, 197, 64)	0	batch_normalization_3[0][0]
max_pooling2d_6 (MaxPooling2D)	(None, 18, 98, 64)	0	activation_52[0][0]
conv2d_8 (Conv2D)	(None, 18, 98, 80)	5120	max_pooling2d_6[0][0]
batch_normalization_4 (BatchNor	(None, 18, 98, 80)	240	conv2d_8[0][0]
activation_53 (Activation)	(None, 18, 98, 80)	0	batch_normalization_4[0][0]
conv2d_9 (Conv2D)	(None, 16, 96, 192)	138240	activation_53[0][0]
batch_normalization_5 (BatchNor	(None, 16, 96, 192)	576	conv2d_9[0][0]
activation_54 (Activation)	(None, 16, 96, 192)	0	batch_normalization_5[0][0]
max_pooling2d_7 (MaxPooling2D)	(None, 7, 47, 192)	0	activation_54[0][0]
conv2d_13 (Conv2D)	(None, 7, 47, 64)	12288	max_pooling2d_7[0][0]
batch_normalization_9 (BatchNor	(None, 7, 47, 64)	192	conv2d_13[0][0]
activation_58 (Activation)	(None, 7, 47, 64)	0	batch_normalization_9[0][0]
conv2d_11 (Conv2D)	(None, 7, 47, 48)	9216	max_pooling2d_7[0][0]
conv2d_14 (Conv2D)	(None, 7, 47, 96)	55296	activation_58[0][0]
batch_normalization_7 (BatchNor	(None, 7, 47, 48)	144	conv2d_11[0][0]
batch_normalization_10 (BatchNo	(None, 7, 47, 96)	288	conv2d_14[0][0]
activation_56 (Activation)	(None, 7, 47, 48)	0	batch_normalization_7[0][0]
activation_59 (Activation)	(None, 7, 47, 96)	0	batch_normalization_10[0][0]
average_pooling2d_1 (AveragePoo	(None, 7, 47, 192)	0	max_pooling2d_7[0][0]
conv2d_10 (Conv2D)	(None, 7, 47, 64)	12288	max_pooling2d_7[0][0]
conv2d_12 (Conv2D)	(None, 7, 47, 64)	76800	activation_56[0][0]
conv2d_15 (Conv2D)	(None, 7, 47, 96)	82944	activation_59[0][0]
conv2d_16 (Conv2D)	(None, 7, 47, 32)	6144	average_pooling2d_1[0][0]
batch_normalization_6 (BatchNor	(None, 7, 47, 64)	192	conv2d_10[0][0]
batch_normalization_8 (BatchNor	(None, 7, 47, 64)	192	conv2d_12[0][0]
batch_normalization_11 (BatchNo	(None, 7, 47, 96)	288	conv2d_15[0][0]
batch_normalization_12 (BatchNo	(None, 7, 47, 32)	96	conv2d_16[0][0]

activation_55 (Activation)	(None, 7, 47, 64)	0	batch_normalization_6[0][0]
activation_57 (Activation)	(None, 7, 47, 64)	0	batch_normalization_8[0][0]
activation_60 (Activation)	(None, 7, 47, 96)	0	batch_normalization_11[0][0]
activation_61 (Activation)	(None, 7, 47, 32)	0	batch_normalization_12[0][0]
mixed0 (Concatenate)	(None, 7, 47, 256)	0	activation_55[0][0]<br>activation_57[0][0]<br>activation_60[0][0]<br>activation_61[0][0]
conv2d_20 (Conv2D)	(None, 7, 47, 64)	16384	mixed0[0][0]
batch_normalization_16 (BatchNo	(None, 7, 47, 64)	192	conv2d_20[0][0]
activation_65 (Activation)	(None, 7, 47, 64)	0	batch_normalization_16[0][0]
conv2d_18 (Conv2D)	(None, 7, 47, 48)	12288	mixed0[0][0]
conv2d_21 (Conv2D)	(None, 7, 47, 96)	55296	activation_65[0][0]
batch_normalization_14 (BatchNo	(None, 7, 47, 48)	144	conv2d_18[0][0]
batch_normalization_17 (BatchNo	(None, 7, 47, 96)	288	conv2d_21[0][0]
activation_63 (Activation)	(None, 7, 47, 48)	0	batch_normalization_14[0][0]
activation_66 (Activation)	(None, 7, 47, 96)	0	batch_normalization_17[0][0]
average_pooling2d_2 (AveragePoo	(None, 7, 47, 256)	0	mixed0[0][0]
conv2d_17 (Conv2D)	(None, 7, 47, 64)	16384	mixed0[0][0]
conv2d_19 (Conv2D)	(None, 7, 47, 64)	76800	activation_63[0][0]
conv2d_22 (Conv2D)	(None, 7, 47, 96)	82944	activation_66[0][0]
conv2d_23 (Conv2D)	(None, 7, 47, 64)	16384	average_pooling2d_2[0][0]
batch_normalization_13 (BatchNo	(None, 7, 47, 64)	192	conv2d_17[0][0]
batch_normalization_15 (BatchNo	(None, 7, 47, 64)	192	conv2d_19[0][0]
batch_normalization_18 (BatchNo	(None, 7, 47, 96)	288	conv2d_22[0][0]
batch_normalization_19 (BatchNo	(None, 7, 47, 64)	192	conv2d_23[0][0]
activation_62 (Activation)	(None, 7, 47, 64)	0	batch_normalization_13[0][0]
activation_64 (Activation)	(None, 7, 47, 64)	0	batch_normalization_15[0][0]
activation_67 (Activation)	(None, 7, 47, 96)	0	batch_normalization_18[0][0]
activation_68 (Activation)	(None, 7, 47, 64)	0	batch_normalization_19[0][0]
mixed1 (Concatenate)	(None, 7, 47, 288)	0	activation_62[0][0]<br>activation_64[0][0]<br>activation_67[0][0]<br>activation_68[0][0]
conv2d_27 (Conv2D)	(None, 7, 47, 64)	18432	mixed1[0][0]
batch_normalization_23 (BatchNo	(None, 7, 47, 64)	192	conv2d_27[0][0]
activation_72 (Activation)	(None, 7, 47, 64)	0	batch_normalization_23[0][0]
conv2d_25 (Conv2D)	(None, 7, 47, 48)	13824	mixed1[0][0]
conv2d_28 (Conv2D)	(None, 7, 47, 96)	55296	activation_72[0][0]
batch_normalization_21 (BatchNo	(None, 7, 47, 48)	144	conv2d_25[0][0]
batch_normalization_24 (BatchNo	(None, 7, 47, 96)	288	conv2d_28[0][0]
activation_70 (Activation)	(None, 7, 47, 48)	0	batch_normalization_21[0][0]
activation_73 (Activation)	(None, 7, 47, 96)	0	batch_normalization_24[0][0]

average_pooling2d_3 (AveragePoo	(None, 7, 47, 288)	0	mixed1[0][0]
conv2d_24 (Conv2D)	(None, 7, 47, 64)	18432	mixed1[0][0]
conv2d_26 (Conv2D)	(None, 7, 47, 64)	76800	activation_70[0][0]
conv2d_29 (Conv2D)	(None, 7, 47, 96)	82944	activation_73[0][0]
conv2d_30 (Conv2D)	(None, 7, 47, 64)	18432	average_pooling2d_3[0][0]
batch_normalization_20 (BatchNo	(None, 7, 47, 64)	192	conv2d_24[0][0]
batch_normalization_22 (BatchNo	(None, 7, 47, 64)	192	conv2d_26[0][0]
batch_normalization_25 (BatchNo	(None, 7, 47, 96)	288	conv2d_29[0][0]
batch_normalization_26 (BatchNo	(None, 7, 47, 64)	192	conv2d_30[0][0]
activation_69 (Activation)	(None, 7, 47, 64)	0	batch_normalization_20[0][0]
activation_71 (Activation)	(None, 7, 47, 64)	0	batch_normalization_22[0][0]
activation_74 (Activation)	(None, 7, 47, 96)	0	batch_normalization_25[0][0]
activation_75 (Activation)	(None, 7, 47, 64)	0	batch_normalization_26[0][0]
mixed2 (Concatenate)	(None, 7, 47, 288)	0	activation_69[0][0]<br>activation_71[0][0]<br>activation_74[0][0]<br>activation_75[0][0]
conv2d_32 (Conv2D)	(None, 7, 47, 64)	18432	mixed2[0][0]
batch_normalization_28 (BatchNo	(None, 7, 47, 64)	192	conv2d_32[0][0]
activation_77 (Activation)	(None, 7, 47, 64)	0	batch_normalization_28[0][0]
conv2d_33 (Conv2D)	(None, 7, 47, 96)	55296	activation_77[0][0]
batch_normalization_29 (BatchNo	(None, 7, 47, 96)	288	conv2d_33[0][0]
activation_78 (Activation)	(None, 7, 47, 96)	0	batch_normalization_29[0][0]
conv2d_31 (Conv2D)	(None, 3, 23, 384)	995328	mixed2[0][0]
conv2d_34 (Conv2D)	(None, 3, 23, 96)	82944	activation_78[0][0]
batch_normalization_27 (BatchNo	(None, 3, 23, 384)	1152	conv2d_31[0][0]
batch_normalization_30 (BatchNo	(None, 3, 23, 96)	288	conv2d_34[0][0]
activation_76 (Activation)	(None, 3, 23, 384)	0	batch_normalization_27[0][0]
activation_79 (Activation)	(None, 3, 23, 96)	0	batch_normalization_30[0][0]
max_pooling2d_8 (MaxPooling2D)	(None, 3, 23, 288)	0	mixed2[0][0]
mixed3 (Concatenate)	(None, 3, 23, 768)	0	activation_76[0][0]<br>activation_79[0][0]<br>max_pooling2d_8[0][0]
conv2d_39 (Conv2D)	(None, 3, 23, 128)	98304	mixed3[0][0]
batch_normalization_35 (BatchNo	(None, 3, 23, 128)	384	conv2d_39[0][0]
activation_84 (Activation)	(None, 3, 23, 128)	0	batch_normalization_35[0][0]
conv2d_40 (Conv2D)	(None, 3, 23, 128)	114688	activation_84[0][0]
batch_normalization_36 (BatchNo	(None, 3, 23, 128)	384	conv2d_40[0][0]
activation_85 (Activation)	(None, 3, 23, 128)	0	batch_normalization_36[0][0]
conv2d_36 (Conv2D)	(None, 3, 23, 128)	98304	mixed3[0][0]
conv2d_41 (Conv2D)	(None, 3, 23, 128)	114688	activation_85[0][0]
batch_normalization_32 (BatchNo	(None, 3, 23, 128)	384	conv2d_36[0][0]

batch_normalization_37 (BatchNo	(None, 3, 23, 128)	384	conv2d_41[0][0]
activation_81 (Activation)	(None, 3, 23, 128)	0	batch_normalization_32[0][0]
activation_86 (Activation)	(None, 3, 23, 128)	0	batch_normalization_37[0][0]
conv2d_37 (Conv2D)	(None, 3, 23, 128)	114688	activation_81[0][0]
conv2d_42 (Conv2D)	(None, 3, 23, 128)	114688	activation_86[0][0]
batch_normalization_33 (BatchNo	(None, 3, 23, 128)	384	conv2d_37[0][0]
batch_normalization_38 (BatchNo	(None, 3, 23, 128)	384	conv2d_42[0][0]
activation_82 (Activation)	(None, 3, 23, 128)	0	batch_normalization_33[0][0]
activation_87 (Activation)	(None, 3, 23, 128)	0	batch_normalization_38[0][0]
average_pooling2d_4 (AveragePoo	(None, 3, 23, 768)	0	mixed3[0][0]
conv2d_35 (Conv2D)	(None, 3, 23, 192)	147456	mixed3[0][0]
conv2d_38 (Conv2D)	(None, 3, 23, 192)	172032	activation_82[0][0]
conv2d_43 (Conv2D)	(None, 3, 23, 192)	172032	activation_87[0][0]
conv2d_44 (Conv2D)	(None, 3, 23, 192)	147456	average_pooling2d_4[0][0]
batch_normalization_31 (BatchNo	(None, 3, 23, 192)	576	conv2d_35[0][0]
batch_normalization_34 (BatchNo	(None, 3, 23, 192)	576	conv2d_38[0][0]
batch_normalization_39 (BatchNo	(None, 3, 23, 192)	576	conv2d_43[0][0]
batch_normalization_40 (BatchNo	(None, 3, 23, 192)	576	conv2d_44[0][0]
activation_80 (Activation)	(None, 3, 23, 192)	0	batch_normalization_31[0][0]
activation_83 (Activation)	(None, 3, 23, 192)	0	batch_normalization_34[0][0]
activation_88 (Activation)	(None, 3, 23, 192)	0	batch_normalization_39[0][0]
activation_89 (Activation)	(None, 3, 23, 192)	0	batch_normalization_40[0][0]
mixed4 (Concatenate)	(None, 3, 23, 768)	0	activation_80[0][0]<br>activation_83[0][0]<br>activation_88[0][0]<br>activation_89[0][0]
conv2d_49 (Conv2D)	(None, 3, 23, 160)	122880	mixed4[0][0]
batch_normalization_45 (BatchNo	(None, 3, 23, 160)	480	conv2d_49[0][0]
activation_94 (Activation)	(None, 3, 23, 160)	0	batch_normalization_45[0][0]
conv2d_50 (Conv2D)	(None, 3, 23, 160)	179200	activation_94[0][0]
batch_normalization_46 (BatchNo	(None, 3, 23, 160)	480	conv2d_50[0][0]
activation_95 (Activation)	(None, 3, 23, 160)	0	batch_normalization_46[0][0]
conv2d_46 (Conv2D)	(None, 3, 23, 160)	122880	mixed4[0][0]
conv2d_51 (Conv2D)	(None, 3, 23, 160)	179200	activation_95[0][0]
batch_normalization_42 (BatchNo	(None, 3, 23, 160)	480	conv2d_46[0][0]
batch_normalization_47 (BatchNo	(None, 3, 23, 160)	480	conv2d_51[0][0]
activation_91 (Activation)	(None, 3, 23, 160)	0	batch_normalization_42[0][0]
activation_96 (Activation)	(None, 3, 23, 160)	0	batch_normalization_47[0][0]
conv2d_47 (Conv2D)	(None, 3, 23, 160)	179200	activation_91[0][0]
conv2d_52 (Conv2D)	(None, 3, 23, 160)	179200	activation_96[0][0]
batch_normalization_43 (BatchNo	(None, 3, 23, 160)	480	conv2d_47[0][0]
batch_normalization_48 (BatchNo	(None, 3, 23, 160)	480	conv2d_52[0][0]

activation_92 (Activation)	(None, 3, 23, 160)	0	batch_normalization_43[0][0]
activation_97 (Activation)	(None, 3, 23, 160)	0	batch_normalization_48[0][0]
average_pooling2d_5 (AveragePoo	(None, 3, 23, 768)	0	mixed4[0][0]
conv2d_45 (Conv2D)	(None, 3, 23, 192)	147456	mixed4[0][0]
conv2d_48 (Conv2D)	(None, 3, 23, 192)	215040	activation_92[0][0]
conv2d_53 (Conv2D)	(None, 3, 23, 192)	215040	activation_97[0][0]
conv2d_54 (Conv2D)	(None, 3, 23, 192)	147456	average_pooling2d_5[0][0]
batch_normalization_41 (BatchNo	(None, 3, 23, 192)	576	conv2d_45[0][0]
batch_normalization_44 (BatchNo	(None, 3, 23, 192)	576	conv2d_48[0][0]
batch_normalization_49 (BatchNo	(None, 3, 23, 192)	576	conv2d_53[0][0]
batch_normalization_50 (BatchNo	(None, 3, 23, 192)	576	conv2d_54[0][0]
activation_90 (Activation)	(None, 3, 23, 192)	0	batch_normalization_41[0][0]
activation_93 (Activation)	(None, 3, 23, 192)	0	batch_normalization_44[0][0]
activation_98 (Activation)	(None, 3, 23, 192)	0	batch_normalization_49[0][0]
activation_99 (Activation)	(None, 3, 23, 192)	0	batch_normalization_50[0][0]
mixed5 (Concatenate)	(None, 3, 23, 768)	0	activation_90[0][0]<br>activation_93[0][0]<br>activation_98[0][0]<br>activation_99[0][0]
conv2d_59 (Conv2D)	(None, 3, 23, 160)	122880	mixed5[0][0]
batch_normalization_55 (BatchNo	(None, 3, 23, 160)	480	conv2d_59[0][0]
activation_104 (Activation)	(None, 3, 23, 160)	0	batch_normalization_55[0][0]
conv2d_60 (Conv2D)	(None, 3, 23, 160)	179200	activation_104[0][0]
batch_normalization_56 (BatchNo	(None, 3, 23, 160)	480	conv2d_60[0][0]
activation_105 (Activation)	(None, 3, 23, 160)	0	batch_normalization_56[0][0]
conv2d_56 (Conv2D)	(None, 3, 23, 160)	122880	mixed5[0][0]
conv2d_61 (Conv2D)	(None, 3, 23, 160)	179200	activation_105[0][0]
batch_normalization_52 (BatchNo	(None, 3, 23, 160)	480	conv2d_56[0][0]
batch_normalization_57 (BatchNo	(None, 3, 23, 160)	480	conv2d_61[0][0]
activation_101 (Activation)	(None, 3, 23, 160)	0	batch_normalization_52[0][0]
activation_106 (Activation)	(None, 3, 23, 160)	0	batch_normalization_57[0][0]
conv2d_57 (Conv2D)	(None, 3, 23, 160)	179200	activation_101[0][0]
conv2d_62 (Conv2D)	(None, 3, 23, 160)	179200	activation_106[0][0]
batch_normalization_53 (BatchNo	(None, 3, 23, 160)	480	conv2d_57[0][0]
batch_normalization_58 (BatchNo	(None, 3, 23, 160)	480	conv2d_62[0][0]
activation_102 (Activation)	(None, 3, 23, 160)	0	batch_normalization_53[0][0]
activation_107 (Activation)	(None, 3, 23, 160)	0	batch_normalization_58[0][0]
average_pooling2d_6 (AveragePoo	(None, 3, 23, 768)	0	mixed5[0][0]
conv2d_55 (Conv2D)	(None, 3, 23, 192)	147456	mixed5[0][0]
conv2d_58 (Conv2D)	(None, 3, 23, 192)	215040	activation_102[0][0]
conv2d_63 (Conv2D)	(None, 3, 23, 192)	215040	activation_107[0][0]

conv2d_64 (Conv2D)	(None, 3, 23, 192)	147456	average_pooling2d_6[0][0]
batch_normalization_51 (BatchNo	(None, 3, 23, 192)	576	conv2d_55[0][0]
batch_normalization_54 (BatchNo	(None, 3, 23, 192)	576	conv2d_58[0][0]
batch_normalization_59 (BatchNo	(None, 3, 23, 192)	576	conv2d_63[0][0]
batch_normalization_60 (BatchNo	(None, 3, 23, 192)	576	conv2d_64[0][0]
activation_100 (Activation)	(None, 3, 23, 192)	0	batch_normalization_51[0][0]
activation_103 (Activation)	(None, 3, 23, 192)	0	batch_normalization_54[0][0]
activation_108 (Activation)	(None, 3, 23, 192)	0	batch_normalization_59[0][0]
activation_109 (Activation)	(None, 3, 23, 192)	0	batch_normalization_60[0][0]
mixed6 (Concatenate)	(None, 3, 23, 768)	0	activation_100[0][0]<br>activation_103[0][0]<br>activation_108[0][0]<br>activation_109[0][0]
conv2d_69 (Conv2D)	(None, 3, 23, 192)	147456	mixed6[0][0]
batch_normalization_65 (BatchNo	(None, 3, 23, 192)	576	conv2d_69[0][0]
activation_114 (Activation)	(None, 3, 23, 192)	0	batch_normalization_65[0][0]
conv2d_70 (Conv2D)	(None, 3, 23, 192)	258048	activation_114[0][0]
batch_normalization_66 (BatchNo	(None, 3, 23, 192)	576	conv2d_70[0][0]
activation_115 (Activation)	(None, 3, 23, 192)	0	batch_normalization_66[0][0]
conv2d_66 (Conv2D)	(None, 3, 23, 192)	147456	mixed6[0][0]
conv2d_71 (Conv2D)	(None, 3, 23, 192)	258048	activation_115[0][0]
batch_normalization_62 (BatchNo	(None, 3, 23, 192)	576	conv2d_66[0][0]
batch_normalization_67 (BatchNo	(None, 3, 23, 192)	576	conv2d_71[0][0]
activation_111 (Activation)	(None, 3, 23, 192)	0	batch_normalization_62[0][0]
activation_116 (Activation)	(None, 3, 23, 192)	0	batch_normalization_67[0][0]
conv2d_67 (Conv2D)	(None, 3, 23, 192)	258048	activation_111[0][0]
conv2d_72 (Conv2D)	(None, 3, 23, 192)	258048	activation_116[0][0]
batch_normalization_63 (BatchNo	(None, 3, 23, 192)	576	conv2d_67[0][0]
batch_normalization_68 (BatchNo	(None, 3, 23, 192)	576	conv2d_72[0][0]
activation_112 (Activation)	(None, 3, 23, 192)	0	batch_normalization_63[0][0]
activation_117 (Activation)	(None, 3, 23, 192)	0	batch_normalization_68[0][0]
average_pooling2d_7<br>(AveragePoo	(None, 3, 23, 768)	0	mixed6[0][0]
conv2d_65 (Conv2D)	(None, 3, 23, 192)	147456	mixed6[0][0]
conv2d_68 (Conv2D)	(None, 3, 23, 192)	258048	activation_112[0][0]
conv2d_73 (Conv2D)	(None, 3, 23, 192)	258048	activation_117[0][0]
conv2d_74 (Conv2D)	(None, 3, 23, 192)	147456	average_pooling2d_7[0][0]
batch_normalization_61 (BatchNo	(None, 3, 23, 192)	576	conv2d_65[0][0]
batch_normalization_64 (BatchNo	(None, 3, 23, 192)	576	conv2d_68[0][0]
batch_normalization_69 (BatchNo	(None, 3, 23, 192)	576	conv2d_73[0][0]
batch_normalization_70 (BatchNo	(None, 3, 23, 192)	576	conv2d_74[0][0]
activation_110 (Activation)	(None, 3, 23, 192)	0	batch_normalization_61[0][0]
activation_113 (Activation)	(None, 3, 23, 192)	0	batch_normalization_64[0][0]

activation_118 (Activation)	(None, 3, 23, 192)	0	batch_normalization_69[0][0]
activation_119 (Activation)	(None, 3, 23, 192)	0	batch_normalization_70[0][0]
mixed7 (Concatenate)	(None, 3, 23, 768)	0	activation_110[0][0]<br>activation_113[0][0]<br>activation_118[0][0]<br>activation_119[0][0]
conv2d_77 (Conv2D)	(None, 3, 23, 192)	147456	mixed7[0][0]
batch_normalization_73 (BatchNo	(None, 3, 23, 192)	576	conv2d_77[0][0]
activation_122 (Activation)	(None, 3, 23, 192)	0	batch_normalization_73[0][0]
conv2d_78 (Conv2D)	(None, 3, 23, 192)	258048	activation_122[0][0]
batch_normalization_74 (BatchNo	(None, 3, 23, 192)	576	conv2d_78[0][0]
activation_123 (Activation)	(None, 3, 23, 192)	0	batch_normalization_74[0][0]
conv2d_75 (Conv2D)	(None, 3, 23, 192)	147456	mixed7[0][0]
conv2d_79 (Conv2D)	(None, 3, 23, 192)	258048	activation_123[0][0]
batch_normalization_71 (BatchNo	(None, 3, 23, 192)	576	conv2d_75[0][0]
batch_normalization_75 (BatchNo	(None, 3, 23, 192)	576	conv2d_79[0][0]
activation_120 (Activation)	(None, 3, 23, 192)	0	batch_normalization_71[0][0]
activation_124 (Activation)	(None, 3, 23, 192)	0	batch_normalization_75[0][0]
conv2d_76 (Conv2D)	(None, 1, 11, 320)	552960	activation_120[0][0]
conv2d_80 (Conv2D)	(None, 1, 11, 192)	331776	activation_124[0][0]
batch_normalization_72 (BatchNo	(None, 1, 11, 320)	960	conv2d_76[0][0]
batch_normalization_76 (BatchNo	(None, 1, 11, 192)	576	conv2d_80[0][0]
activation_121 (Activation)	(None, 1, 11, 320)	0	batch_normalization_72[0][0]
activation_125 (Activation)	(None, 1, 11, 192)	0	batch_normalization_76[0][0]
max_pooling2d_9 (MaxPooling2D)	(None, 1, 11, 768)	0	mixed7[0][0]
mixed8 (Concatenate)	(None, 1, 11, 1280)	0	activation_121[0][0]<br>activation_125[0][0]<br>max_pooling2d_9[0][0]
conv2d_85 (Conv2D)	(None, 1, 11, 448)	573440	mixed8[0][0]
batch_normalization_81 (BatchNo	(None, 1, 11, 448)	1344	conv2d_85[0][0]
activation_130 (Activation)	(None, 1, 11, 448)	0	batch_normalization_81[0][0]
conv2d_82 (Conv2D)	(None, 1, 11, 384)	491520	mixed8[0][0]
conv2d_86 (Conv2D)	(None, 1, 11, 384)	1548288	activation_130[0][0]
batch_normalization_78 (BatchNo	(None, 1, 11, 384)	1152	conv2d_82[0][0]
batch_normalization_82 (BatchNo	(None, 1, 11, 384)	1152	conv2d_86[0][0]
activation_127 (Activation)	(None, 1, 11, 384)	0	batch_normalization_78[0][0]
activation_131 (Activation)	(None, 1, 11, 384)	0	batch_normalization_82[0][0]
conv2d_83 (Conv2D)	(None, 1, 11, 384)	442368	activation_127[0][0]
conv2d_84 (Conv2D)	(None, 1, 11, 384)	442368	activation_127[0][0]
conv2d_87 (Conv2D)	(None, 1, 11, 384)	442368	activation_131[0][0]
conv2d_88 (Conv2D)	(None, 1, 11, 384)	442368	activation_131[0][0]
average_pooling2d_8 (AveragePoo	(None, 1, 11, 1280)	0	mixed8[0][0]

conv2d_81 (Conv2D)	(None, 1, 11, 320)	409600	mixed8[0][0]
batch_normalization_79 (BatchNo	(None, 1, 11, 384)	1152	conv2d_83[0][0]
batch_normalization_80 (BatchNo	(None, 1, 11, 384)	1152	conv2d_84[0][0]
batch_normalization_83 (BatchNo	(None, 1, 11, 384)	1152	conv2d_87[0][0]
batch_normalization_84 (BatchNo	(None, 1, 11, 384)	1152	conv2d_88[0][0]
conv2d_89 (Conv2D)	(None, 1, 11, 192)	245760	average_pooling2d_8[0][0]
batch_normalization_77 (BatchNo	(None, 1, 11, 320)	960	conv2d_81[0][0]
activation_128 (Activation)	(None, 1, 11, 384)	0	batch_normalization_79[0][0]
activation_129 (Activation)	(None, 1, 11, 384)	0	batch_normalization_80[0][0]
activation_132 (Activation)	(None, 1, 11, 384)	0	batch_normalization_83[0][0]
activation_133 (Activation)	(None, 1, 11, 384)	0	batch_normalization_84[0][0]
batch_normalization_85 (BatchNo	(None, 1, 11, 192)	576	conv2d_89[0][0]
activation_126 (Activation)	(None, 1, 11, 320)	0	batch_normalization_77[0][0]
mixed9_0 (Concatenate)	(None, 1, 11, 768)	0	activation_128[0][0]<br>activation_129[0][0]
concatenate_1 (Concatenate)	(None, 1, 11, 768)	0	activation_132[0][0]<br>activation_133[0][0]
activation_134 (Activation)	(None, 1, 11, 192)	0	batch_normalization_85[0][0]
mixed9 (Concatenate)	(None, 1, 11, 2048)	0	activation_126[0][0]<br>mixed9_0[0][0]<br>concatenate_1[0][0]<br>activation_134[0][0]
conv2d_94 (Conv2D)	(None, 1, 11, 448)	917504	mixed9[0][0]
batch_normalization_90 (BatchNo	(None, 1, 11, 448)	1344	conv2d_94[0][0]
activation_139 (Activation)	(None, 1, 11, 448)	0	batch_normalization_90[0][0]
conv2d_91 (Conv2D)	(None, 1, 11, 384)	786432	mixed9[0][0]
conv2d_95 (Conv2D)	(None, 1, 11, 384)	1548288	activation_139[0][0]
batch_normalization_87 (BatchNo	(None, 1, 11, 384)	1152	conv2d_91[0][0]
batch_normalization_91 (BatchNo	(None, 1, 11, 384)	1152	conv2d_95[0][0]
activation_136 (Activation)	(None, 1, 11, 384)	0	batch_normalization_87[0][0]
activation_140 (Activation)	(None, 1, 11, 384)	0	batch_normalization_91[0][0]
conv2d_92 (Conv2D)	(None, 1, 11, 384)	442368	activation_136[0][0]
conv2d_93 (Conv2D)	(None, 1, 11, 384)	442368	activation_136[0][0]
conv2d_96 (Conv2D)	(None, 1, 11, 384)	442368	activation_140[0][0]
conv2d_97 (Conv2D)	(None, 1, 11, 384)	442368	activation_140[0][0]
average_pooling2d_9 (AveragePoo	(None, 1, 11, 2048)	0	mixed9[0][0]
conv2d_90 (Conv2D)	(None, 1, 11, 320)	655360	mixed9[0][0]
batch_normalization_88 (BatchNo	(None, 1, 11, 384)	1152	conv2d_92[0][0]
batch_normalization_89 (BatchNo	(None, 1, 11, 384)	1152	conv2d_93[0][0]
batch_normalization_92 (BatchNo	(None, 1, 11, 384)	1152	conv2d_96[0][0]
batch_normalization_93 (BatchNo	(None, 1, 11, 384)	1152	conv2d_97[0][0]
conv2d_98 (Conv2D)	(None, 1, 11, 192)	393216	average_pooling2d_9[0][0]
batch_normalization_86 (BatchNo	(None, 1, 11, 320)	960	conv2d_90[0][0]

activation_137 (Activation)	(None, 1, 11, 384)	0	batch_normalization_88[0][0]
activation_138 (Activation)	(None, 1, 11, 384)	0	batch_normalization_89[0][0]
activation_141 (Activation)	(None, 1, 11, 384)	0	batch_normalization_92[0][0]
activation_142 (Activation)	(None, 1, 11, 384)	0	batch_normalization_93[0][0]
batch_normalization_94 (BatchNo	(None, 1, 11, 192)	576	conv2d_98[0][0]
activation_135 (Activation)	(None, 1, 11, 320)	0	batch_normalization_86[0][0]
mixed9_1 (Concatenate)	(None, 1, 11, 768)	0	activation_137[0][0]<br>activation_138[0][0]
concatenate_2 (Concatenate)	(None, 1, 11, 768)	0	activation_141[0][0]<br>activation_142[0][0]
activation_143 (Activation)	(None, 1, 11, 192)	0	batch_normalization_94[0][0]
mixed10 (Concatenate)	(None, 1, 11, 2048)	0	activation_135[0][0]<br>mixed9_1[0][0]<br>concatenate_2[0][0]<br>activation_143[0][0]
flatten_4 (Flatten)	(None, 22528)	0	mixed10[0][0]
dense_7 (Dense)	(None, 128)	2883712	flatten_4[0][0]
dense_8 (Dense)	(None, 3)	387	dense_7[0][0]
Total params: 24,686,883<br>Trainable params: 2,884,099<br>Non-trainable params: 21,802,784			

3. Tools used in the experiment

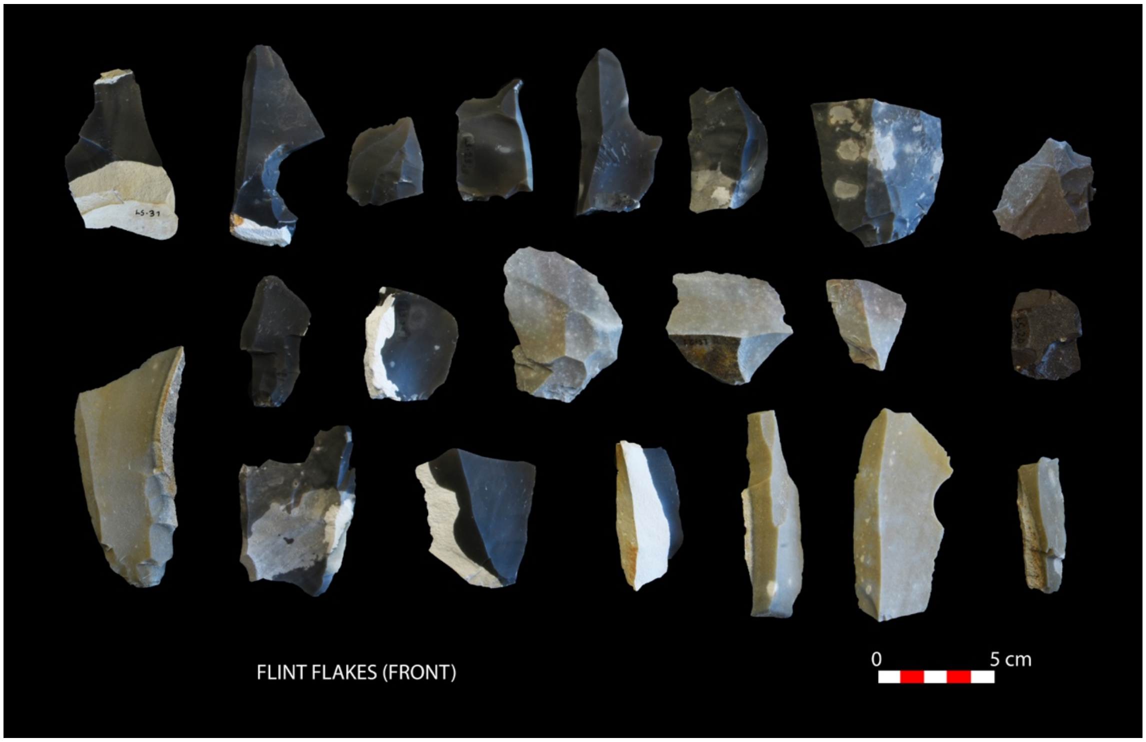

3.1. Dorsal side of flakes used in the experiment

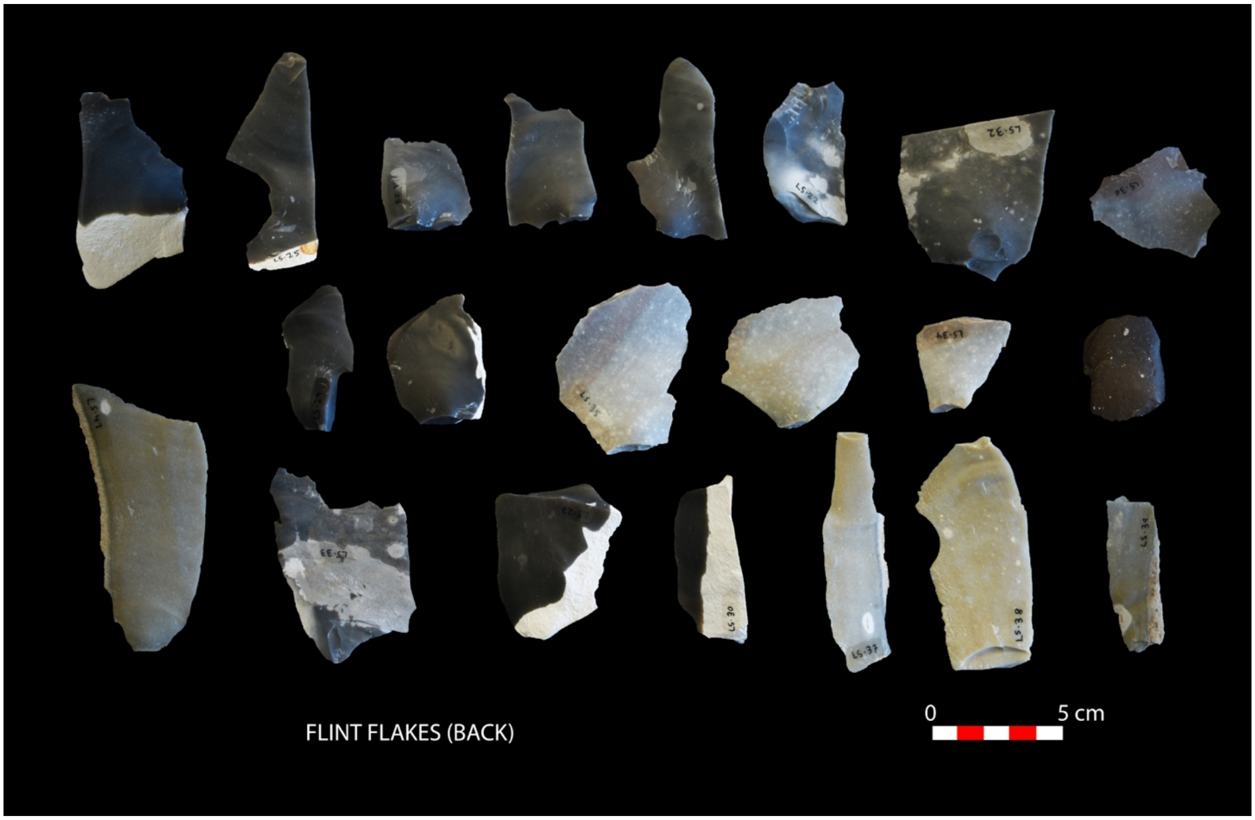

3.2. Ventral side of the flakes used in the experiment.

References

1. L. Bourgeon, A. Burke, T. Higham, Earliest Human Presence in North America Dated to the Last Glacial Maximum: New Radiocarbon Dates from Bluefish Caves, Canada. *PLoS One* **12**, e0169486 (2017).

2. M. C. Arriaza, M. Domínguez-Rodrigo, J. Yravedra, E. Baquedano, Lions as Bone Accumulators? Paleontological and Ecological Implications of a Modern Bone Assemblage from Olduvai Gorge. *PLoS One* **11**, e0153797 (2016).

3. J. Yravedra, L. Lagos, F. Bárcena, A taphonomic study of wild wolf (Canis lupus) modification of horse bones in Northwestern Spain. *Journal of Taphonomy* **9**, 37–65 (2011).

4. S. P. McPherron, *et al.*, Evidence for stone-tool-assisted consumption of animal tissues before 3.39 million years ago at Dikika, Ethiopia. *Nature* **466**, 857–860 (2010).

5. M. Domínguez-Rodrigo, T. R. Pickering, H. T. Bunn, Configurational approach to identifying the earliest hominin butchers. *Proc. Natl. Acad. Sci. U. S. A.* **107**, 20929–20934 (2010).

6. S. P. McPherron, *et al.*, Tool-marked bones from before the Oldowan change the paradigm. *Proc. Natl. Acad. Sci. U. S. A.* **108**, E116; author reply E117 (2011).

7. M. Domínguez-Rodrigo, T. R. Pickering, H. T. Bunn, Experimental study of cut marks made with rocks unmodified by human flaking and its bearing on claims of 3.4-million-year-old butchery evidence from Dikika, Ethiopia. *J. Archaeol. Sci.* **39**, 205–214 (2012).

8. M. Domínguez-Rodrigo, T. R. Pickering, H. T. Bunn, Reply to McPherron et al.: Doubting Dikika is about data, not paradigms. *Proceedings of the National Academy of Sciences* **108**, E117–E117 (2011).

9. S. Harmand, *et al.*, 3.3-million-year-old stone tools from Lomekwi 3, West Turkana, Kenya. *Nature* **521**, 310–315 (2015).

10. M. Domínguez-Rodrigo, L. Alcalá, 3.3-Million-year-old stone tools and butchery traces? More evidence needed. *PaleoAnthropology* **2016**, 46–53 (2016).

11. J. E. Lewis, S. Harmand, An earlier origin for stone tool making: implications for cognitive evolution and the transition to Homo. *Philos. Trans. R. Soc. Lond. B Biol. Sci.* **371** (2016).

12. M. Dominguez-Rodrigo, L. Alcalá, Pliocene Archaeology at Lomekwi 3? New Evidence Fuels More Skepticism. *Journal of African Archaeology* **17**, 173–176 (2019).

13. D. Gommery, *et al.*, Les plus anciennes traces d'activités anthropiques de Madagascar sur des ossements d'hippopotames subfossiles d'Anjohibe (Province de Mahajanga). *Comptes Rendus Palevol* **10**, 271–278 (2011).

14. V. R. Perez, *et al.*, Evidence of early butchery of giant lemurs in Madagascar. *J. Hum. Evol.* **49**, 722–742 (2005).

15. S. M. Goodman, W. L. Jungers, *Extinct Madagascar: Picturing the Island's Past* (University of Chicago Press, 2014).

16. A. Anderson, *et al.*, New evidence of megafaunal bone damage indicates late colonization of Madagascar. *PLoS One* **13**, e0204368 (2018).

17. J. Hansford, *et al.*, Early Holocene human presence in Madagascar evidenced by exploitation of avian megafauna. *Sci Adv* **4**, eaat6925 (2018).

18. B. Martínez-Navarro, "Early Pleistocene Faunas of Eurasia and Hominin Dispersals" in *Out of Africa I: The First Hominin Colonization of Eurasia*, J. G. Fleagle, J. J. Shea, F. E. Grine,

A. L. Baden, R. E. Leakey, Eds. (Springer Netherlands, 2010), pp. 207–224.

19. B. M. Navarro, P. Palmqvist, Presence of the African MachairodontMegantereon whitei (Broom, 1937)(Felidae, Carnivora, Mammalia) in the Lower Pleistocene Site of Venta Micena (Orce, Granada, Spain), with some Considerations on the Origin, Evolution and Dispersal of the Genus. *J. Archaeol. Sci.* **22**, 569–582 (1995).

20. E. Carbonell, *et al.*, Early hominid dispersals: A technological hypothesis for "out of Africa." *Quat. Int.* **223-224**, 36–44 (2010).

21. P. Palmqvist, *et al.*, The giant hyena Pachycrocuta brevirostris: Modelling the bone-cracking behavior of an extinct carnivore. *Quat. Int.* **243**, 61–79 (2011).

22. A. Arribas, P. Palmqvist, On the Ecological Connection Between Sabre-tooths and Hominids: Faunal Dispersal Events in the Lower Pleistocene and a Review of the Evidence for the First Human Arrival in Europe. *Journal of Archaeological Science* **26**, 571–585 (1999).

23. D. Joubert, Hunting behaviour of lions (Panthera leo) on elephants (Loxodonta africana) in the Chobe National Park, Botswana. *Afr. J. Ecol.* **44**, 279–281 (2006).

24. R. John Power, R. X. Shem Compion, Lion predation on elephants in the Savuti, Chobe National Park, Botswana. *Afr. Zool.* **44**, 36–44 (2009).

25. C. J. McBRIDE, Age and size categories of lion prey in Chobe National Park, Botswana. *Botsw. Notes Rec.* **16**, 139–143 (1984).

26. H. P. Andreassen, G. Neo-Mahupeleng, Ø. Flagstad, P. Wegge, The Chobe Riverfront Lion Population: A Large Predator as Responder to Elephant-Induced Habitat Heterogeneity. *Elephants and Savanna Woodland Ecosystems*, 251–267 (2014).

27. G. B. Schaller, *The Serengeti Lion: A Study of Predator-Prey Relations* (University of Chicago Press, 2009).

28. D. R. Prothero, *The Princeton Field Guide to Prehistoric Mammals* (Princeton University Press, 2016).

29. J. A. Meachen-Samuels, Morphological convergence of the prey-killing arsenal of sabertooth predators. *Paleobiology* **38**, 1–14 (2012).

30. W. D. Turnbull, W. Segall, The ear region of the marsupial sabertooth,Thylacosmilus: Influence of the sabertooth lifestyle upon it, and convergence with placental sabertooths. *Journal of Morphology* **181**, 239–270 (1984).

31. M. Antón, *Sabertooth* (Indiana University Press, 2013).

32. B. Van Valkenburgh, C. B. Ruff, Canine tooth strength and killing behaviour in large carnivores. *Journal of Zoology* **212**, 379–397 (1987).

33. H. N. Bryant, A. P. Russell, J. J. Thomason, Carnassial functioning in nimravid and felid sabertooths: theoretical basis and robustness of inferences. *Functional morphology in vertebrate paleontology*, 116–135 (1995).

34. C. W. Marean, C. L. Ehrhardt, Paleoanthropological and paleoecological implications of the taphonomy of a sabertooth's den. *J. Hum. Evol.* **29**, 515–547 (1995).

35. A. Hartstone-Rose, S. Wahl, Using radii-of-curvature for the reconstruction of extinct South African carnivoran masticatory behavior. *C. R. Palevol* **7**, 629–643 (2008).

36. A. Hartstone-Rose, Reconstructing the diets of extinct South African carnivorans from premolar "intercuspid notch" morphology. *Journal of Zoology* **285**, 119–127 (2011).

37. L. R. G. Desantis, B. W. Schubert, J. R. Scott, P. S. Ungar, Implications of diet for the extinction of saber-toothed cats and American lions. *PLoS One* **7**, e52453 (2012).

38. L. R. G. DeSantis, B. W. Schubert, E. Schmitt-Linville, Dental microwear textures of carnivorans from the La Brea Tar Pits, California and potential extinction implications. *Contributions in Science* (2015).

39. M. P. Espigares, *et al.*, The earliest cut marks of Europe: a discussion on hominin subsistence patterns in the Orce sites (Baza basin, SE Spain). *Scientific Reports* **9** (2019).

40. M. P. Espigares, *et al.*, Homo vs. Pachycrocuta: Earliest evidence of competition for an elephant carcass between scavengers at Fuente Nueva-3 (Orce, Spain). *Quat. Int.* **295**, 113–125 (2013).
